# Thymidine phosphorylase and prostrate cancer cell proliferation inhibitory activities of synthetic 4-hydroxybenzohydrazides: *In vitro*, kinetic, and *in silico* studies

**DOI:** 10.1371/journal.pone.0227549

**Published:** 2020-01-27

**Authors:** Sumaira Javaid, Syed Muhammad Saad, Humaira Zafar, Rizwana Malik, Khalid Mohammed Khan, M. Iqbal Choudhary, Atta-ur Rahman

**Affiliations:** 1 Dr. Panjwani Center for Molecular Medicine and Drug Research, International Center of Chemical and Biological Sciences, University of Karachi, Karachi, Pakistan; 2 H. E. J., Research Institute of Chemistry, International Center for Chemical and Biological Sciences, University of Karachi, Karachi, Pakistan; 3 Department of Chemistry, University of Karachi, Karachi, Pakistan; 4 Department of Clinical Pharmacy, Institute for Research and Medical Consultations (IRMC), Imam Abdulrahman Bin Faisal University, Dammam, Saudi Arabia; 5 Department of Biochemistry, Faculty of Sciences, King Abdulaziz University, Jeddah, Saudi Arabia; Duke University School of Medicine, UNITED STATES

## Abstract

Over-expression of thymidine phosphorylase (TP) plays a key role in many pathological complications, including angiogenesis which leads to cancer cells proliferation. Thus in search of new anticancer agents, a series of 4-hydroxybenzohydrazides (**1–29**) was synthesized, and evaluated for *in vitro* thymidine phosphorylase inhibitory activity. Twenty compounds **1**–**3**, **6**–**14**, **16**, **19**, **22**–**24**, and **27**–**29** showed potent to weak TP inhibitory activities with IC_50_ values in the range of 6.8 to 229.5 *μ*M, in comparison to the standards *i*.*e*. tipiracil (IC_50_ = 0.014 ± 0.002 *μ*M) and 7-deazaxanthine (IC_50_ = 41.0 ± 1.63 *μ*M). Kinetic studies on selected inhibitors **3**, **9**, **14**, **22**, **27**, and **29** revealed uncompetitive and non-competitive modes of inhibition. Molecular docking studies of these inhibitors indicated that they were able to interact with the amino acid residues present in allosteric site of TP, including Asp391, Arg388, and Leu389. Antiproliferative (cytotoxic) activities of active compounds were also evaluated against mouse fibroblast (3T3) and prostate cancer (PC3) cell lines. Compounds **1**, **2**, **19**, and **22**–**24** exhibited anti-proliferative activities against PC3 cells with IC_50_ values between 6.5 to 10.5 *μ*M, while they were largely non-cytotoxic to 3T3 (mouse fibroblast) cells proliferation. Present study thus identifies a new class of dual inhibitors of TP and cancer cell proliferation, which deserves to be further investigated for anti-cancer drug development.

## Introduction

Thymidine phosphorylase (TP) (EC 2.4.2.4) is an enzyme of pyrimidine salvage pathway, primarily responsible for maintaining nucleotide homeostasis. TP Over-expression has been reported in several pathological conditions, including rheumatoid arthritis, chronic inflammatory diseases, psoriasis, and tumor angiogenesis. Neoplastic tissues of bladder, gastric, cervical, lung, colon, prostate, esophageal, and breast cancers show hyperactivity of TP. Over-expression of TP in tumor cell lines is an indication of its role in angiogenesis [1]. Role of TP as angiogenic enzyme was proposed to be identical to human platelet derived endothelial cell growth factor (PD-ECGF), which promotes angiogenesis by facilitating endothelial cell proliferation, and migration [2]. TP Enzyme is present in many cells and tissues, such as platelets, stromal cells, macrophages, endothelial cells, reticulocytes, glial cells, and ovary [1].

TP Comprises of two identical subunits, with molecular weight between 90 to 110 kDa in *Escherichia coli* and mammals, respectively. Each subunit consists of a large *α*/*β* domain and a smaller *α*-helical domain, separated from each other by a cavity [3]. Active site of TP comprises of thymidine and phosphate binding sites. In addition to this, it also has a hydrophobic pocket near the substrate binding sites. In the presence of inorganic phosphate, it catalyzes the reversible cleavage of glycosidic bond of pyrimidine 2′-deoxynucleotides to 2′-deoxyribose-1-phosphate. Deoxyribose-1-phosphate then undergoes dephosphorylation to produce 2-D-deoxyribose, which is used either as energy source for the cell or secreted out of the cell where it may act as an angiogenic growth factor [3, 4].

Significant efforts have been focused on the development of TP inhibitors with possible therapeutic potential since 1960’s. Some compounds were identified with excellent TP inhibitory activity, and tested pre-clinically and clinically. However, only one drug (Lonsurf^®^) is in clinical use for the management of metastatic colorectal cancer. This drug was approved by US-FDA in 2015 and is combination of tipiracil (TP inhibitor) and trifluridine (a cytotoxin). This drugis associated with adverse side effects which demands the identification of new leads capable of inhibiting TP enzyme and thereby TP associated pathologies [5]. 7-Deazaxanthine (7-DX) and 6-amino-5-bromouracil (6A5BU) are widely used as reference compounds for *in vitro* studies against the TP enzyme [1, 6].

**Fig 1:**
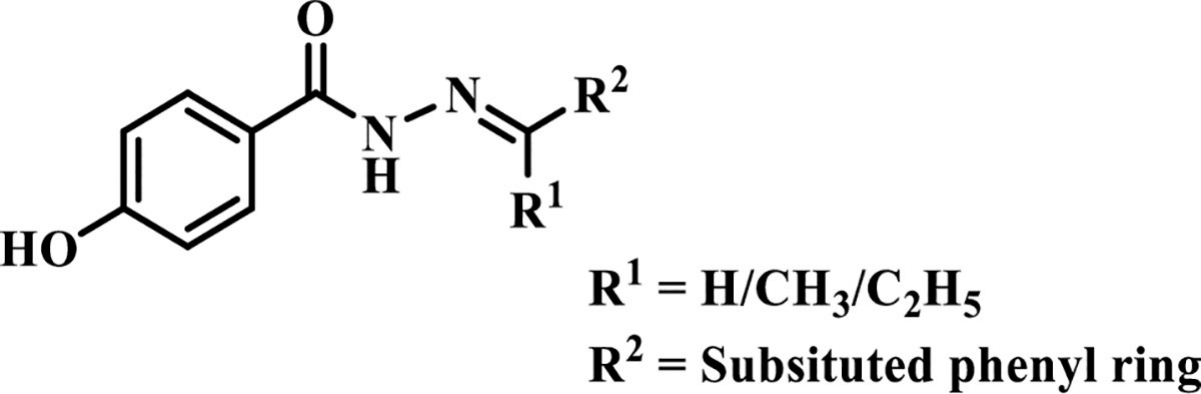
Skeleton of 4-hydroxybenzohydrazide: 4-hydroxybenzohydrazide derivatives 1–29.

Our research group has reported several classes of synthetic and natural compounds as TP inhibitors, such as 3-formylchromones [7], 1,3,4-oxadiazoles [8], 2-arylquinazolin-4(3*H*)-ones [9], thymidine esters [10], plant glycosides [11], and salirepin derivatives from *Symplocos racemosa* [12]. In continuation of this, we evaluated 4-hydroxylbenzohydarzide derivatives for their TP inhibitory activity (Fig 1). Schiff bases and hydrazones are reported to possess a wide variety of biological properties, such as anticonvulsant, antibacterial, antihypertensive, anti-inflammatory, antifungal, anticancer, antipyretic, antimicrobial, cytotoxic, anti-HIV, and herbicidal activities [13, 14]. All compounds were known previously [15–29], except **7**, **16**, **27**, and **29** which were identified as new analogues.

Twenty-nine derivatives of 4-hydroxybenzohydarzide were subjected to an *in vitro* spectrophotometric TP inhibition assay. Some of the most active compounds were then subjected to kinetic and molecular docking studies in order to determine their mechanism of inhibition of TP enzyme. TP is particularly reported to be over-expressed in the prostate cancer, therefore, active compounds against TP were also evaluated for their effect on the proliferation of prostate cancer cells (PC3) using the (3-[4,5-dimethylthiazole-2-yl]-2,5-diphenyl-tetrazolium bromide) MTT colorimetric assay [2, 30, 31]. Interestingly, some of these compounds were also able to inhibit the PC3 cancer cells proliferation. Present study therefore identifies dual inhibitors of TP, and cancer cell proliferation.

## Material and methods

Enzyme thymidine phosphorylase (*E*. *coli*, EC 2.4.2.4), thymidine, Dulbecco's Modified Eagle Medium (DMEM), cyclohexamide, and doxorubicin were purchased from Sigma Aldrich, USA. Standard inhibitors 7-deazaxanthine and tipiracil were purchased from Santa Cruz Biotechnology Inc., USA. Deionized water used for buffer preparation was collected from Simplicity Water Purification System (Milipore). 0.25% Trypsin EDTA was purchased from Gibco, Invitrogen, New Zealand, fetal bovine serum (FBS) was purchased from A & E Scientific (PAA), USA, 0.4% Trypan Blue solution was purchased from Amersco, USA, and 3-(4,5-dimethythiazol-2-yl)-2,5-diphenyl tetrazolium bromide (MTT) was purchased from MP Biomedicals, France. 4-Hydroxybenzohydrazide, substituted benzaldehydes, ethanol and acetic acid were purchased from TCI, Japan. All reagents were of analytical grade and used directly without purification.

^1^H-NMR Spectra were recorded on Avance Bruker AM 300 and 400 MHz instruments. Electron Impact Mass Spectrometric (EI-MS) experiments were run on Finnigan MAT-311A (Germany) mass spectrometer.

### Cell lines

Mouse fibroblast cell line (NIH/3T3 (ATCC^®^ CRL-1658^™^)), and prostate cancer line (PC3 (ATCC® CRL-1435™)) were purchased from American Type Culture Collection (ATCC), USA. As we have purchased these lines directly from ATCC, therefore we did not perform any quality control testing procedures.

### Thymidine phosphorylase inhibition assay

Since human TP is not commercially available, we used commercially available recombinant TP (expressed in *E*. *coli*) enzyme. Primary sequence of TP is mostly conserved throughout the evolution and mammalian TP share 39% sequence similarity with TP of *E*. *coli*. The mammalian enzyme also shares 65–70% similarity with the active site residues of *E*. *coli* TP enzyme [2].

Assay for TP inhibition was performed spectrophotometrically, following the method of Bera *et al*. with some modifications [32]. Enzyme TP catalyzed the reversible phosphorolysis of thymidine to thymine and 2-deoxyribose-1-phosphate. Reaction mixture contained 150 *μ*L of potassium phosphate buffer (pH 7.0, 50 mM), 20 *μ*L of enzyme (0.058 U/well), and 10 *μ*L of test compound (0.5 mM in dimethylsulfoxide). The reaction mixture was incubated for 10 min at 30°C. 20 *μ*L of substrate (1.5 mM) was then added and change in absorbance was recorded after every minute for a total time period of 10 minutes at 290 nm in ELISA plate reader (Spectra Max-340, Molecular Devices, CA, USA). Tipiracil and 7-deazaxanthine were used as standard inhibitors in this assay.

We have used the 96-well UV-plates, purchased from Corning (Catalogue No. COSTAR 3635, Lot No. 00912050). In addition to this, we also carried out pre-read (blank reference) on a solution that does not contain the substrate, and this gave us zero reading. We then added the substrate (thymidine), and recorded the final readings. Therefore, the chances of getting erroneous results were minimized.

### Anti-proliferative assay

Anti-proliferative activity of active compounds was evaluated by using the standard MTT (3-[4,5-dimethylthiazole-2-yl]-2,5-diphenyl-tetrazolium bromide) colorimetric assay in 96-well plate [33].

It is a colorimetric assay that measures the reduction of MTT by mitochondrial enzyme *i*.*e*. succinate dehydrogenase. The MTT enters into the mitochondria of cell, where it is reduced to an insoluble formazan salt. As reduction of MTT can only occur in metabolically active cells, the level of activity is actually a measure of the viability of the cells [34].

Mouse fibroblast normal cell line 3T3, and prostate cancer line (PC3) were cultured separately in DMEM, supplemented with 5% of FBS, 100 IU/mL of penicillin and 100 *μ*g/mL of streptomycin, and kept at 37°C in 5% CO_2_ incubator. For the preparation of cell culture, 100 *μ*L/well of cell solution (5 x 10^4^ cells/mL) was added into 96-well plate. The plate was incubated for overnight, and fresh medium was added after the removal of old medium. The test compounds were also added in different concentrations into the plate, and again incubated for 48 h. After the completion of this incubation period, 200 *μ*L MTT (0.5 mg/mL) was added, and plate was again incubated for 4 h, after this final incubation, 100 *μ*L of DMSO was added to each well. The level of MTT reduction to formazan was evaluated by change in absorbance at 540 nm for 3T3, and 570 nm for PC3 using a micro-plate reader (Spectra Max plus, Molecular Devices, CA, USA). The anti-proliferative activity was recorded as concentration of the inhibitor causing 50% growth inhibition (IC_50_) of cells.

### Protocol for kinetic studies

Kinetic studies were carried out to identify the mechanism of inhibition by these compounds. Inhibitor could bind with the enzyme in multiple ways, such as in competitive, non-competitive, mixed or uncompetitive way. In kinetic assay, the enzyme (0.058 U/200 *μ*L) was incubated with different concentrations of inhibitor for 10 min at 30°C. The reaction was then initiated by adding different concentrations (0.1875–1.5 mM) of substrate (thymidine), and the resulting degradation of thymidine was measured continuously at 290 nm for 10 min on ELISA plate reader. Every experiment was run in triplicate.

Lineweaver-Burk plot was plotted to determine the type of inhibition. This was accomplished by plotting the reciprocal of the rate of reaction against the reciprocal of the substrate concentration. *K*i values were determined by secondary re-plot of Lineweaver-Burk plot, and reconfirmed by Dixon plot.

### Molecular docking studies

Molecular docking studies were carried out in order to understand the interaction of inhibitors (ligands) with TP (receptors). The structure of TP was taken from PDB (4LHM) which is the crystallographic structure of TP in *E*. *coli*. Protein was prepared using Protein Preparation Wizard and OPLS3 force field [35,36] in order to add missing hydrogens. The sulphate ions were replaced by phosphate so they did not affect the conformation of protein and occupy the same position as that of sulphate. Ligands were prepared to get the correct ionization and protonation states using *Ligprep* module [37] in Maestro Schrӧdinger2018-1.

Since all the inhibitors showed non- and uncompetitive mode of inhibition in kinetic studies, site map analysis [38,39] was performed to find out the best allosteric site available in TP. Five allosteric sites were observed and the one with highest score *i*.*e*. **1** was finally selected for the docking studies of these ligands. The dimensions of grid box were 15 Ǻ x 15 Ǻ x 15 Ǻ from the mass of centre of each docked ligand and extra precision mode of Glide [40–43] was used for the docking of ligands.

### Statistical analysis

Results obtained for the enzyme inhibitory activity and MTT assay were analysed using SoftMax Pro 4.8 software (Molecular Devices, CA, USA) and Microsoft Excel. Percent of enzyme inhibition, and growth inhibition of cells was calculated using the following formula:

PercentInhibition=100‐(Absorbanceoftest/Absorbanceofcontrol)x100

Results were presented as means ± standard error mean from triplicate (*n* = 3) observation. IC_50_ values were determined by using EZ-FIT, Enzyme kinetics software by Perrella Scientific, Inc., USA. Grafit 7.0 version was used to determine the kinetics parameters. The software was purchased from the Erithacus Software Ltd. (Wilmington House, West Sussex RH19 3AU, UK).

### General procedure for the synthesis of compounds 1–29

In a typical procedure, 4-hydroxylbenzohydrazones (**1**–**29**) were synthesized by mixing 4-hydroxylbenzohydrazide (1.5 mmol), substituted benzaldehydes (1.5 mmol) in ethanol (20 mL) with a catalytic amount of acetic acid (1 mL). The mixture was refluxed for 3 h, while progress of the reaction was monitored through thin layer chromatography. After completion of reaction, the reaction mixture was poured into China dish to let the solvent evaporate slowly at room temperature to afford crystals of the products. Structures of the compounds were deduced by using NMR and mass spectroscopic techniques.

#### 4-Hydroxyl-*N'*-[(*E*)-(2^′^-hydroxylphenyl)methylidene]benzohydrazide (1)

Yield: 85.7%; ^1^H NMR: (400 MHz, DMSO-*d*_6_): *δ* 11.90 (s, 1H, NH), 11.40 (s, 1H, 2^′^-OH), 10.16 (s, 1H, 4-OH), 8.58 (s, 1H, N = CH), 7.82 (d, 2H, *J*_2,3_ = *J*_6,5_ = 8.4 Hz, H-2, H-6), 7.50 (dd, 1H, *J*_6′,5′_ = 8.0 Hz, *J*_6′,4′_ = 1.2 Hz, H-6^′^), 7.30 (m, 1H, H-4^′^), 6.92 (m, 1H, H-5^′^), 6.90 (m, 1H, H-3^′^), 6.87 (d, 2H, *J*_3,2_ = *J*_5,6_ = 8.8 Hz, H-3, H-5); EI-MS: *m/z* (rel. abund. %), 256 (M^+^, 22), 137 (80), 121 (100), 93 (31); Anal. Calcd for C_14_H_12_N_2_O_3_: C, 65.62; H, 4.72; N, 10.93; O, 18.73; Found: C, 65.60; H, 4.75; N, 10.98.

#### *N'*-[(*E*)-(2^′^,3^′^-Dihydroxylphenyl)methylidene]-4-hydroxylbenzohydrazide (2)

Yield: 80.7%; ^1^HNMR: (400 MHz, DMSO-*d*_6_): *δ* 11.89 (s, 1H, NH), 11.30 (s, 1H, 2^′^-OH), 10.15 (s, 1H, 4-OH), 9.13 (s, 1H, 3^′^-OH), 8.53 (s, 1H, N = CH), 7.82 (d, 2H, *J*_2,3_ = *J*_6,5_ = 8.4 Hz, H-2,H-6), 6.92 (dd, 1H, *J*_6′,5′_ = 8.0 Hz, *J*_6′,4′_ = 1.2 Hz, H-6^′^), 6.87 (d, 2H, *J*_3,2_ = *J*_5,6_ = 8.4 Hz, H-3, H-5), 6.84 (d, 1H, *J*_4′,5′_ = 8.0 Hz, H-4^′^), 6.74 (t, 1H, *J*_5′(4′,6′)_ = 7.6 Hz, H-5^′^); EI-MS: *m/z* (rel. abund. %), 272 (M^+^, 64), 137 (28), 121 (100), 93 (32); Anal. Calcd for C_14_H_12_N_2_O_4_: C, 61.76; H, 4.44; N, 10.29; O, 23.51; Found: C, 61.78; H, 4.45; N, 10.35.

#### *N'*-[(*E*)-(3^′^,4^′^-Dihydroxylphenyl)methylidene]-4-hydroxylbenzohydrazide (3)

Yield: 81.7%; ^1^HNMR: (400 MHz, DMSO-*d*_6_): *δ* 11.36 (s, 1H, NH), 10.05 (br s, 1H, 4-OH), 9.27 (br s, 2H, 4^′^-OH, 3^′^-OH), 8.21 (s, 1H, N = CH), 7.77 (d, 2H, *J*_2,3_ = *J*_6,5_ = 8.4 Hz, H-2, H-6), 7.21 (s, 1H, H-2^′^), 6.9 (d, 2H, *J*_3,2_ = *J*_5,6_ = 7.6 Hz, H-3, H-5), 6.84 (d, 1H, *J*_6′,5′_ = 8.4 Hz, H-6^′^), 6.77 (d, 1H, *J*_5′,6′_ = 8.0 Hz, H-5^′^); EI-MS: *m/z* (rel. abund. %), 272 (M^+^, 8), 137 (27), 121 (100), 93 (21); Anal. Calcd for C_14_H_12_N_2_O_4_: C, 61.76; H, 4.44; N, 10.29; O, 23.51; Found: C, 61.75; H, 4.40; N, 10.30.

#### 4-Hydroxyl-*N'*-[(*E*)-(2^′^,4^′^,6^′^-trihydroxylphenyl)methylidene]benzohydrazide (4)

Yield: 84.5%; ^1^HNMR: (400 MHz, DMSO-*d*_6_): *δ* 11.64 (s, 1H, NH), 11.08 (s, 2H, 2^′^-OH, 6^′^-OH), 10.09 (s, 1H, 4-OH), 9.74 (s, 1H, 4^′^-OH), 8.75 (s, 1H, N = CH), 7.79 (d, 2H, *J*_2,3_ = *J*_6,5_ = 8.4 Hz, H-2, H-6), 6.85 (d, 2H, *J*_3,2_ = *J*_5,6_ = 8.4 Hz, H-3, H-5), 5.82 (s, 2H, H-3^′^, H-5^′^); EI-MS: *m/z* (rel. abund. %), 288 (M^+^, 3), 152 (16), 137 (6), 121 (100), 93 (41); Anal. Calcd for C_14_H_12_N_2_O_5_: C, 58.33; H, 4.20; N, 9.72; O, 27.75; Found: C, 58.30; H, 4.25; N, 9.73.

#### 4-Hydroxyl-*N'*-[(*E*)-(2^′^,3^′^,4^′^-trihydroxylphenyl)methylidene]benzohydrazide (5)

Yield: 75.0%; ^1^HNMR: (400 MHz, DMSO-*d*_6_): *δ* 11.72 (s, 1H, NH), 11.64 (s, 1H, 2^′^-OH), 10.12 (s, 1H, 4-OH), 9.39 (s, 1H, 4^′^-OH), 8.44 (s, 1H, 3^′^-OH), 8.40 (s, 1H, N = CH), 7.80 (d, 2H, *J*_2,3_ = *J*_6,5_ = 8.8 Hz, H-2, H-6), 6.86 (d, 2H, *J*_3,2_ = *J*_5,6_ = 8.8 Hz, H-3, H-5), 6.74 (d, 1H, *J*_5′,6′_ = 8.4 Hz, H-5^′^), 6.38 (d, 1H, *J*_6′,5′_ = 8.4 Hz, H-6^′^); EI-MS: *m/z* (rel. abund. %), 288 (M^+^, 73), 151 (8), 137 (23), 121 (100), 93 (37); Anal. Calcd for C_14_H_12_N_2_O_5_: C, 58.33; H, 4.20; N, 9.72; O, 27.75; Found: C, 58.35; H, 4.21; N, 9.74.

#### 4-Hydroxyl-*N'*-[(*E*)-(3^′^-hydroxylphenyl)methylidene]benzohydrazide (6)

Yield: 84.6%; ^1^H NMR: (400 MHz, DMSO-*d*_6_): *δ* 11.54 (s, 1H, NH), 10.08 (s, 1H, 4-OH), 9.58 (s, 1H, 3^′^-OH), 8.32 (s, 1H, N = CH), 7.79 (d, 2H, *J*_2,3_ = *J*_6,5_ = 8.8 Hz, H-2, H-6), 7.25 (t, 1H, *J*_5′(4′,6′)_ = 7.6 Hz, H-5^′^), 7.16 (s, 1H, H-2^′^), 7.07 (d, 1H, *J*_6′,5′_ = 7.6 Hz, H-6^′^), 6.85 (d, 2H, *J*_3,2_ = *J*_5,6_ = 8.8 Hz, H-3, H-5), 6.81 (dd, 1H, *J*_4′,5′_ = 8.0 Hz, *J*_4′,6′_ = 1.6 Hz, H-4^′^); EI-MS: *m/z* (rel. abund. %), 256 (M^+^, 8), 163 (2), 137 (89), 121 (100), 93 (41); Anal. Calcd for C_14_H_12_N_2_O_3_: C, 65.62; H, 4.72; N, 10.93; O, 18.73; Found: C, 65.65; H, 4.76; N, 10.96.

#### 4-Hydroxyl-*N'*-[(*E*)-(2^′^,4^′^,5^′^-trihydroxylphenyl)methylidene]benzohydrazide (7)

Yield: 78.7%; ^1^HNMR: (400 MHz, DMSO-*d*_6_): *δ* 11.57 (s, 1H, NH), 10.73 (s, 1H, 2^′^-OH), 10.09 (s, 1H, 4-OH), 9.50 (s, 1H, 4^′^-OH), 8.51 (s, 1H, 5^′^-OH), 8.38 (s, 1H, N = CH), 7.8 (d, 2H, *J*_2,3_ = *J*_6,5_ = 8.8 Hz, H-2, H-6), 6.85 (d, 2H, *J*_3,2_ = *J*_5,6_ = 8.8 Hz, H-3, H-5), 6.82 (s, 1H, H-6^′^), 6.31 (s, 1H, H-3^′^); EI-MS: *m/z* (rel. abund. %), 288 (M^+^, 7), 152 (5), 151 (15), 137 (24), 121 (100), 93 (11); Anal. Calcd for C_14_H_12_N_2_O_5_: C, 58.33; H, 4.20; N, 9.72; O, 27.75; Found: C, 58.35; H, 4.23; N, 9.70.

#### 4-Hydroxyl-*N'*-[(*E*)-(4^′^-hydroxylphenyl)methylidene]benzohydrazide (8)

Yield: 90.0%; ^1^H NMR: (400 MHz, DMSO-*d*_6_): *δ* 11.40 (s, 1H, NH), 9.96 (br s, 2H, 4-OH, 4^′^-OH), 8.30 (s, 1H, N = CH), 7.78 (d, 2H, *J*_2,3_ = *J*_6,5_ = 8.8 Hz, H-2, H-6), 7.53 (d, 2H, *J*_2′,3′_ = *J*_6′,5′_ = 8.0 Hz, H-2^′^, H-6^′^), 6.84 (d, 2H, *J*_3,2_ = *J*_5,6_ = 6.0 Hz, H-3, H-5), 6.82 (d, 2H, *J*_3′,2′_ = *J*_5′,6′_ = 5.6 Hz, H-3,H-5^′^); EI-MS: *m/z* (rel. abund. %), 256 (M^+^, 13), 137 (70), 121 (100), 93 (23); Anal. Calcd for C_14_H_12_N_2_O_3_: C, 65.62; H, 4.72; N, 10.93; O, 18.73; Found: C, 65.64; H, 4.70; N, 10.90.

#### *N'*-[(*E*)-(4^′^-Chlorophenyl)methylidene]-4-hydroxylbenzohydrazide (9)

Yield: 89.4%; ^1^HNMR: (400 MHz, DMSO-*d*_6_): *δ* 11.67 (s, 1H, NH), 10.11 (s, 1H, 4-OH), 8.40 (s, 1H, N = CH), 7.80 (d, 2H, *J*_2,3_ = *J*_6,5_ = 8.8 Hz, H-2, H-6), 7.72 (d, 2H, *J*_2′,3′_ = *J*_6′,5′_ = 8.8 Hz, H-2^′^, H-6^′^), 7.51 (d, 2H, *J*_3′,2′_ = *J*_5′,6′_ = 8.4 Hz, H-3^′^, H-5^′^), 6.86 (d, 2H, *J*_3,2_ = *J*_5,6_ = 8.8 Hz, H-3, H-5); EI-MS: *m/z* (rel. abund. %), 276 (M^+^+2, 2), 274 (M^+^, 4), 137 (37), 121 (100), 111 (4), 93 (25); Anal. Calcd for C_14_H_11_ClN_2_O_2_: C, 61.21; H, 4.04; Cl, 12.90; N, 10.20; O, 11.65; Found: C, 61.20; H, 4.07; N, 10.20.

#### 4-Hydroxyl-*N'*-{(*E*)-[4^′^-(methoxy)phenyl]methylidene}benzohydrazide (10)

Yield: 77.8%; ^1^HNMR: (300 MHz, DMSO-*d*_6_): *δ* 11.47 (s, 1H, NH), 10.06 (s, 1H, 4-OH), 8.35 (s, 1H, N = CH), 7.79 (d, 2H, *J*_2,3_ = *J*_6,5_ = 8.7 Hz, H-2, H-6), 7.65 (d, 2H, *J*_2′,3′_ = *J*_6′,5′_ = 8.1 Hz, H-2^′^, H-6^′^), 7.02 (d, 2H, *J*_3′,2′_ = *J*_5′,6′_ = 8.7 Hz, H-3^′^, H-5^′^), 6.85 (d, 2H, *J*_3,2_ = *J*_5,6_ = 8.7 Hz, H-3, H-5), 3.79 (s, 3H, 4^′^-OCH_3_); EI-MS: *m/z* (rel. abund. %), 270 (M^+^, 14), 150 (55), 137 (80), 121 (100), 93 (13); Anal. Calcd for C_15_H_14_N_2_O_3_: C, 66.66; H, 5.22; N, 10.36; O, 17.76; Found: C, 66.65; H, 5.20; N, 10.37.

#### *N'*-[(*E*)-(3^′^-Chlorophenyl)methylidene]-4-hydroxylbenzohydrazide (11)

Yield: 80.3%; ^1^HNMR: (400 MHz, DMSO-*d*_6_): *δ* 11.74 (s, 1H, NH), 10.12 (s, 1H, 4-OH), 8.39 (s, 1H, N = CH), 7.80 (d, 2H, *J*_2,3_ = *J*_6,5_ = 8.8 Hz, H-2, H-6), 7.74 (s, 1H, H-2^′^), 7.65 (s, 1H, H-6^′^), 7.47 (s, 1H, H-4^′^), 7.46 (s, 1H, H-5^′^), 6.86 (d, 2H, *J*_3,2_ = *J*_5,6_ = 8.4 Hz, H-3, H-5); EI-MS: *m/z* (rel. abund. %), 276 (M^+^+2, 3), 274 (M^+^, 8), 137 (95), 121 (100), 111 (3), 93 (44); Anal. Calcd for C_14_H_11_ClN_2_O_2_: C, 61.21; H, 4.04; Cl, 12.90; N, 10.20; O, 11.65; Found: C, 61.23; H, 4.05; N, 10.21.

#### 4-Hydroxyl-*N'*-{(*E*)-[2^′^-(methoxy)phenyl]methylidene}benzohydrazide (12)

Yield: 71.9%; ^1^HNMR: (400 MHz, DMSO-*d*_6_): *δ* 11.61 (s, 1H, NH), 10.07 (s, 1H, 4-OH), 8.76 (s, 1H, N = CH), 7.85 (d, 1H, *J*_6′,5′_ = 6.8 Hz, H-6^′^), 7.81 (d, 2H, *J*_2,3_ = *J*_6,5_ = 8.8 Hz, H-2, H-6), 7.41 (m, 1H, H-4^′^), 7.10 (d, 1H, *J*_3′,4′_ = 8.4 Hz, H-3^′^), 7.02 (m, 1H, H-5^′^), 6.84 (d, 2H, *J*_3,2_ = *J*_5,6_ = 8.8 Hz, H-3, H-5), 3.85 (s, 3H, 2^′^-OCH_3_); EI-MS: *m/z* (rel. abund. %), 270 (M^+^, 14), 137 (100), 121 (100), 119 (68), 93 (67); Anal. Calcd for C_15_H_14_N_2_O_3_: C, 66.66; H, 5.22; N, 10.36; O, 17.76; Found: C, 66.68; H, 5.24; N, 10.39.

#### *N'*-{(*E*)-[3^′^,4^′^-*Bis*(methoxy)phenyl]methylidene}-4-hydroxylbenzohydrazide (13)

Yield: 71.7%; ^1^HNMR: (400 MHz, DMSO-*d*_6_): *δ* 11.49 (s, 1H, NH), 10.08 (br s, 1H, 4-OH), 8.34 (s, 1H, N = CH), 7.79 (d, 2H, *J*_2,3_ = *J*_6,5_ = 8.4 Hz, H-2, H-6), 7.31 (s, 1H, H-2^′^), 7.17 (d, 1H, *J*_6′,5′_ = 8.0 Hz, H-6^′^), 7.02 (d, 1H, *J*_5′,6′_ = 8.4 Hz, H-5^′^), 6.85 (d, 2H, *J*_3,2_ = *J*_5,6_ = 8.4 Hz, H-3, H-5), 3.80 (s, 3H, 4^′^-OCH_3_), 3.79 (s, 3H, 3^′^-OCH_3_); EI-MS: *m/z* (rel. abund. %), 300 (M^+^, 23), 163 (99), 148 (6), 137 (16), 121 (100), 93 (11); Anal. Calcd for C_16_H_16_N_2_O_4_: C, 63.99; H, 5.37; N, 9.33; O, 21.31; Found: C, 63.97; H, 5.39; N, 9.34.

#### *N'*-[(*E*)-(4^′^-Bromophenyl)methylidene]-4-hydroxylbenzohydrazide (14)

Yield: 81.9%; ^1^HNMR: (400 MHz, DMSO-*d*_6_): *δ* 11.68 (s, 1H, NH), 10.11 (s, 1H, 4-OH), 8.38 (s, 1H, N = CH), 7.80 (d, 2H, *J*_2,3_ = *J*_6,5_ = 8.4 Hz, H-2, H-6), 7.64 (s, 4H, H-2^′^, H-6^′^, H-3^′^, H-5^′^), 6.86 (d, 2H, *J*_3,2_ = *J*_5,6_ = 8.4 Hz, H-3, H-5); EI-MS: *m/z* (rel. abund. %), 320 (M^+^+2, 5), 318 (M^+^, 22), 181 (12), 163 (2), 155 (4), 137 (83), 121 (100), 93 (29); Anal. Calcd for C_14_H_11_BrN_2_O_2_: C, 52.69; H, 3.47; Br, 25.04; N, 8.78; O, 10.03; Found: C, 52.67; H, 3.49; N, 8.75.

#### *N'*-[(*E*)-(2^′^,4^′^-Dichlorophenyl)methylidene]-4-hydroxylbenzohydrazide (15)

Yield: 65.7%; ^1^HNMR: (400 MHz, DMSO-*d*_6_): *δ* 11.90 (s, 1H, NH), 10.15 (s, 1H, 4-OH), 8.77 (s, 1H, N = CH), 8.00 (d, 1H, *J*_6′,5′_ = 7.6 Hz, H-6^′^), 7.82 (d, 2H, *J*_2,3_ = *J*_6,5_ = 8.8 Hz, H-2, H-6), 7.70 (d, 1H, *J*_3′,5′_ = 2 Hz, H-3^′^), 7.52 (dd, 1H, *J*_5′,6′_ = 8.8 Hz, *J*_5′,3′_ = 2.0 Hz, H-5^′^), 6.86 (d, 2H, *J*_3,2_ = *J*_5,6_ = 8.8 Hz, H-3, H-5); EI-MS: *m/z* (rel. abund. %), 312 (M^+^+4, 1), 310 (M^+^+2, 1), 308 (M^+^, 2), 137 (55), 121 (100), 93 (17); Anal. Calcd for C_14_H_10_Cl_2_N_2_O_2_: C, 54.39; H, 3.26; Cl, 22.93; N, 9.06; O, 10.35; Found: C, 54.37; H, 3.28; N, 9.08.

#### 4-Hydroxyl-*N'*-[(*E*)-(2^′^-methylphenyl)methylidene]benzohydrazide (16)

Yield: 81.3%; ^1^HNMR: (400 MHz, DMSO-*d*_6_): *δ* 11.59 (s, 1H, NH), 10.10 (s, 1H, 4-OH), 8.71 (s, 1H, N = CH), 7.80 (d, 2H, *J*_2,3_ = *J*_6,5_ = 8.4 Hz, H-2, H-6), 7.78 (s, 1H, H-6^′^), 7.29 (m, 3H, H-4^′^, H-5^′^, H-3^′^), 6.86 (d, 2H, *J*_3,2_ = *J*_5,6_ = 8.4 Hz, H-3, H-5), 2.49 (s, 3H, 2^′^-CH_3_); EI-MS: *m/z* (rel. abund. %), 254 (M^+^, 3), 137 (100), 121 (100), 93 (34); Anal. Calcd for C_15_H_14_N_2_O_2_: C, 70.85; H, 5.55; N, 11.02; O, 12.58; Found: C, 70.87; H, 5.58; N, 11.01.

#### *N'*-{(*E*)-[4^′^-(Dimethylamino)phenyl]methylidene}4hydroxylbenzohydrazide (17)

Yield: 86.0%; ^1^HNMR: (300 MHz, DMSO-*d*_6_): *δ* 11.31 (s, 1H, NH), 10.04 (br s, 1H, 4-OH), 8.27 (s, 1H, N = CH), 7.78 (d, 2H, *J*_2,3_ = *J*_6,5_ = 8.4 Hz, H-2, H-6), 7.52 (d, 2H, *J*_2′,3′_ = *J*_6′,5′_ = 8.4 Hz, H-2^′^, H-6^′^), 6.84 (d, 2H, *J*_3′,2′_ = *J*_5′,6′_ = 8.7 Hz, H-3^′^, H-5^′^), 6.75 (d, 2H, *J*_3,2_ = *J*_5,6_ = 8.7 Hz, H-3, H-5), 2.96 (s, 6H, 4^′^-N(CH_3_)_2_); EI-MS: *m/z* (rel. abund. %), 283 (M^+^, 89), 163 (73), 146 (100), 137 (7), 121 (85), 93 (27); Anal. Calcd for C_16_H_17_N_3_O_2_: C, 67.83; H, 6.05; N, 14.83; O, 11.29; Found: C, 67.85; H, 6.07; N, 14.81.

#### 4-Hydroxyl-*N'*-{(*E*)-[4^′^-(methylsulfanyl)phenyl]methylidene}benzohydrazide (18)

Yield: 75.2%; ^1^HNMR: (400 MHz, DMSO-*d*_6_): *δ* 11.56 (s, 1H, NH), 10.08 (s, 1H, 4-OH), 8.36 (s, 1H, N = CH), 7.79 (d, 2H, *J*_2,3_ = *J*_6,5_ = 8.8 Hz, H-2, H-6), 7.63 (d, 2H, *J*_2′,3′_ = *J*_6′,5′_ = 8.0 Hz, H-2^′^, H-6^′^), 7.32 (d, 2H, *J*_3′,2′_ = *J*_5′,6′_ = 8.4 Hz, H-3^′^, H-5^′^), 6.85 (d, 2H, *J*_3,2_ = *J*_5,6_ = 8.8 Hz, H-3, H-5), 2.53 (s, 3H, 4^′^-SCH_3_); EI-MS: *m/z* (rel. abund. %), 286 (M^+^, 31), 149 (88), 137 (79), 121 (100), 93 (74); Anal. Calcd for C_15_H_14_N_2_O_2_S: C, 62.92; H, 4.93; N, 9.78; O, 11.17; S, 11.20; Found: C, 62.90; H, 4.91; N, 9.75.

#### 4-Hydroxyl-*N*'-[(*E*)-(2^′^-hydroxyl-3^′^-methoxyphenyl)methylidene]benzohydrazide (19)

Yield: 82.2%; ^1^HNMR: (400 MHz, DMSO-*d*_6_): *δ* 11.86 (br s, 1H, NH), 11.13 (br s, 1H, 2^′^-OH), 10.15 (br s, 1H, 4-OH), 8.59 (s, 1H, N = CH), 7.82 (d, 2H, *J*_2,3_ = *J*_6,5_ = 8.8 Hz, H-2, H-6), 7.10 (dd, 1H, *J*_6′,5′_ = 7.6 Hz, *J*_6′,4′_ = 0.8 Hz, H-6^′^), 7.02 (d, 1H, *J*_4′,5′_ = 7.6 Hz, H-4^′^), 6.87 (d, 2H, *J*_3,2_ = *J*_5,6_ = 8.8 Hz, H-3, H-5), 6.82 (m, 1H, H-5^′^), 3.80 (s, 3H, 3^′^-OCH_3_); EI-MS: *m/z* (rel. abund. %), 286 (M^+^, 61), 149 (23), 137 (52), 121 (100), 93 (38); Anal. Calcd for C_15_H_14_N_2_O_4_: C, 62.93; H, 4.93; N, 9.79; O, 22.35; Found: C, 62.90; H, 4.95; N, 9.77.

#### *N'*-[(*E*)-(5^′^-Bromo-2^′^-hydroxylphenyl)methylidene]-4-hydroxylbenzohydrazide (20)

Yield: 61.9%; ^1^HNMR: (300 MHz, DMSO-*d*_6_): *δ* 11.97 (s, 1H, NH), 11.40 (s, 1H, 2^′^-OH), 10.15 (s, 1H, 4-OH), 8.55 (s, 1H, N = CH), 7.82 (d, 2H, *J*_2,3_ = *J*_6,5_ = 8.4 Hz, H-2, H-6), 7.75 (s, 1H, H-6^′^), 7.42 (dd, 1H, *J*_4′,3′_ = 8.7 Hz, *J*_4′,6′_ = 2.1 Hz, H-4^′^), 6.90 (m, 1H, H-3^′^), 6.87 (d, 2H, *J*_3,2_ = *J*_5,6_ = 8.4 Hz, H-3, H-5); EI-MS: *m/z* (rel. abund. %), 336 (M^+^+2, 34), 334 (M^+^, 37), 197 (4), 137 (97), 121 (100), 93 (54); Anal. Calcd for C_14_H_11_BrN_2_O_3_: C, 50.17; H, 3.31; Br, 23.84; N, 8.36; O, 14.32; Found: C, 50.15; H, 3.33; N, 8.35.

#### *N'*-[(*E*)-(5^′^-Chloro-2^′^-hydroxylphenyl)methylidene]-4-hydroxylbenzohydrazide (21)

Yield: 75.6%; ^1^HNMR: (300 MHz, DMSO-*d*_6_): *δ* 11.98 (s, 1H, NH), 11.39 (s, 1H, 2^′^-OH), 10.16 (s, 1H, 4-OH), 8.56 (s, 1H, N = CH), 7.82 (d, 2H, *J*_2,3_ = *J*_6,5_ = 8.4 Hz, H-2, H-6), 7.62 (s, 1H, H-6^′^), 7.31 (dd, 1H, *J*_4′,3′_ = 8.7 Hz, *J*_4′,6′_ = 2.7 Hz, H-4^′^), 6.95 (d, 1H, *J*_3′,4′_ = 8.7 Hz, H-3^′^), 6.87 (d, 2H, *J*_3,2_ = *J*_5,6_ = 8.7 Hz, H-3, H-5); EI-MS: *m/z* (rel. abund. %), 292 (M^+^+2, 21), 290 (M^+^, 60), 153 (7), 137 (62), 121 (100), 93 (56); Anal. Calcd for C_14_H_11_ClN_2_O_3_: C, 57.84; H, 3.81; Cl, 12.19; N, 9.64; O, 16.51; Found: C, 57.81; H, 3.84; N, 9.65.

#### *N'*-[(*E*)-(3^′^,5^′^-Dibromo-2^′^-hydroxylphenyl)methylidene]-4-hydroxylbenzohydrazide (22)

Yield: 57.3%; ^1^HNMR: (400 MHz, DMSO-*d*_6_): *δ* 12.85 (s, 1H, NH), 12.31 (s, 1H, 2^′^-OH), 10.22 (s, 1H, 4-OH), 8.48 (s, 1H, N = CH), 7.84 (d, 2H, *J*_2,3_ = *J*_6,5_ = 8.4 Hz, H-2, H-6), 7.80 (d, 1H, *J*_6′,4′_ = 2.0 Hz, H-6^′^), 7.78 (s, 1H, H-4^′^), 6.89 (d, 2H, *J*_3,2_ = *J*_5,6_ = 8.4 Hz, H-3, H-5); EI-MS: *m/z* (rel. abund. %), 416 (M^+^+4, 18), 414 (M^+^+2, 50), 412 (M^+^, 21), 275 (5), 137 (100), 121 (100), 93 (67); Anal. Calcd for C_14_H_10_Br_2_N_2_O_3_: C, 40.61; H, 2.43; Br, 38.60; N, 6.77; O, 11.59; Found: C, 40.63; H, 2.45; N, 6.79.

#### 4-Hydroxyl-*N'*-{(*E*)-[2^′^-hydroxyl-5^′^-(methoxy)phenyl]methylidene}benzo-hydrazide (23)

Yield: 69.4%; ^1^HNMR: (300 MHz, DMSO-*d*_6_): *δ* 11.88 (s, 1H, NH), 10.79 (s, 1H, 2^′^-OH), 10.23 (br s, 1H, 4-OH), 8.56 (s, 1H, N = CH), 7.82 (d, 2H, *J*_2,3_ = *J*_6,5_ = 8.4 Hz, H-2, H-6), 7.08 (d, 1H, *J*_6′,4′_ = 2.7 Hz, H-6^′^), 6.91 (m, 2H, H-4^′^, H-3^′^), 6.87 (d, 2H, *J*_3,2_ = *J*_5,6_ = 8.4 Hz, H-3, H-5), 3.72 (s, 3H, 5^′^-OCH_3_); EI-MS: *m/z* (rel. abund. %), 286 (M^+^, 68), 269 (2), 149 (26), 137 (34), 121 (100), 93 (38); Anal. Calcd for C_15_H_14_N_2_O_4_: C, 62.93; H, 4.93; N, 9.79; O, 22.35; Found: C, 62.95; H, 4.92; N, 9.78.

#### *N'*-{(*E*)-[3^′^-Ethoxy-2^′^-hydroxylphenyl]methylidene}-4-hydroxylbenzohydrazide (24)

Yield: 55.9%; ^1^HNMR: (400 MHz, DMSO-*d*_6_): *δ* 11.87 (s, 1H, NH), 11.14 (s, 1H, 2^′^-OH), 10.15 (s, 1H, 4-OH), 8.58 (s, 1H, N = CH), 7.82 (d, 2H, *J*_2,3_ = *J*_6,5_ = 8.4 Hz, H-2, H-6), 7.09 (d, 1H, *J*_6′,5′_ = 8.0 Hz, H-6^′^), 7.01 (d, 1H, *J*_4′,5′_ = 7.6 Hz, H-4^′^), 6.87 (m, 1H, H-5^′^), 6.82 (d, 2H, *J*_3,2_ = *J*_5,6_ = 8.0 Hz, H-3, H-5), 4.07 (q, 2H, *J CH*_*2*_. *CH*_*3*_ = 13.6 Hz, 6.8 Hz, CH_2_), 1.35 (t, 3H, *J CH*_*3*_,*CH*_*2*_ = 7.2 Hz, CH_3_); EI-MS: *m/z* (rel. abund. %), 300 (M^+^, 54), 285 (2), 163 (23), 137 (30), 121 (100), 93 (32); Anal. Calcd for C_16_H_16_N_2_O_4_: C, 63.99; H, 5.37; N, 9.33; O, 21.31; Found: C, 63.97; H, 5.35; N, 9.35.

#### 4-Hydroxyl-*N'*-[(*E*)-1-(2^′^-hydroxylphenyl)ethylidene]benzohydrazide (25)

Yield: 73.5%; ^1^HNMR: (400 MHz, DMSO-*d*_6_): *δ* 13.40 (s, 1H, NH), 11.03 (s, 1H, 2^′^-OH), 10.14 (s, 1H, 4-OH), 7.83 (d, 2H, *J*_2,3_ = *J*_6,5_ = 8.8 Hz, H-2, H-6), 7.62 (d, 1H, *J*_6′,5′_ = 7.6 Hz, H-6^′^), 7.29 (t, 1H, *J*_4′(5′,3′)_ = 7.2 Hz, H-4^′^), 6.89 (s, 2H, H-3^′^, H-5^′^), 6.88 (d, 2H, *J*_3,2_ = *J*_5,6_ = 8.4 Hz, H-3, H-5), 2.49 (s, 3H, -CH_3_); EI-MS: *m/z* (rel. abund. %), 270 (M^+^, 75), 255 (42), 177 (2), 150 (54), 121 (100), 93 (39); Anal. Calcd for C_15_H_14_N_2_O_3_: C, 66.66; H, 5.22; N, 10.36; O, 17.76; Found: C, 66.68; H, 5.24; N, 10.35.

#### *N'*-[(*E*)-1-(2^′^,4^′^-Dihydroxylphenyl)ethylidene]-4-hydroxylbenzohydrazide (26)

Yield: 83.9%; ^1^HNMR: (400 MHz, DMSO-*d*_6_): *δ* 13.57 (s, 1H, NH), 10.85 (s, 1H, 2^′^-OH), 10.1 (s, 1H, 4-OH), 9.79 (s, 1H, 4^′^-OH), 7.80 (d, 2H, *J*_2,3_ = *J*_6,5_ = 8.4 Hz, H-2, H-6), 7.42 (d, 1H, *J*_6′,5′_ = 8.8 Hz, H-6^′^), 6.86 (d, 2H, *J*_3,2_ = *J*_5,6_ = 8.4 Hz, H-3, H-5), 6.32 (dd, 1H, *J*_5′,6′_ = 8.8 Hz, *J*_5′,3′_ = 1.6 Hz, H-5^′^), 6.25 (d, 1H, *J*_3′,5′_ = 2.0 Hz, H-3^′^), 2.37 (s, 3H, -CH_3_); EI-MS: *m/z* (rel. abund. %), 286 (M^+^, 16), 271 (8), 151 (15), 137 (24), 121 (100), 93 (16); Anal. Calcd for C_15_H_14_N_2_O_4_: C, 62.93; H, 4.93; N, 9.79; O, 22.35; Found: C, 62.95; H, 4.91; N, 9.77.

#### *N'*-[(*E*)-1-(2^′^,6^′^-Dihydroxylphenyl)ethylidene]-4-hydroxylbenzohydrazide (27)

Yield: 76.4%; ^1^HNMR: (300 MHz, DMSO-*d*_6_): *δ* 12.62 (s, 1H, NH), 10.97 (br s, 1H, 2^′^-OH), 10.13 (br s, 1H, 4-OH), 8.87 (s, 1H, 6^′^-OH), 7.82 (d, 2H, *J*_2,3_ = *J*_6,5_ = 8.4 Hz, H-2, H-6), 6.95 (d, 1H, *J*_4′,3′_ = *J*_4′,5′_ = 9.0 Hz, H-4^′^), 6.87 (d, 2H, *J*_3,2_ = *J*_5,6_ = 8.7 Hz, H-3, H-5), 6.72 (d, 2H, *J*_3′(4′,5′)_ = *J*_5′(4′,3′)_ = 7.2 Hz, H-3^′^, H-5^′^), 2.38 (s, 3H, -CH_3_); EI-MS: *m/z* (rel. abund. %), 286 (M^+^, 63), 271 (5), 121 (100), 93 (22); Anal. Calcd for C_15_H_14_N_2_O_4_: C, 62.93; H, 4.93; N, 9.79; O, 22.35; Found: C, 62.92; H, 4.92; N, 9.80.

#### *N'*-[(*E*)-1-(2^′^,5^′^-Dihydroxylphenyl)ethylidene]-4-hydroxylbenzohydrazide (28)

Yield: 79.3%; ^1^HNMR: (300 MHz, DMSO-*d*_6_): *δ* 12.62 (s, 1H, NH), 10.97 (br s, 1H, 2^′^-OH), 10.15 (br s, 1H, 4-OH), 8.87 (s, 1H, 5^′^-OH), 7.82 (d, 2H, *J*_2,3_ = *J*_6,5_ = 8.7 Hz, H-2, H-6), 6.94 (s, 1H, H-6^′^), 6.87 (d, 2H, *J*_3′,4′_ = *J*_4′,3′_ = 8.4 Hz, H-3^′^, H-4^′^), 6.75 (d, 2H, *J*_3,2_ = *J*_5,6_ = 8.7 Hz, H-3, H-5), 2.38 (s, 3H, CH_3_); EI-MS: *m/z* (rel. abund. %), 286 (M^+^, 50), 269 (8), 166 (38), 121 (100), 93 (10); Anal. Calcd for C_15_H_14_N_2_O_4_: C, 62.93; H, 4.93; N, 9.79; O, 22.35; Found: C, 62.92; H, 4.91; N, 9.76.

#### *N'*-[(*E*)-1-(2^′^,5^′^-Dihydroxylphenyl)propylidene]-4-hydroxylbenzohydrazide (29)

Yield: 49.0%; ^1^HNMR: (400 MHz, DMSO-*d*_6_): *δ* 12.68 (s, 1H, NH), 11.03 (s, 1H, 2^′^-OH), 10.13 (s, 1H, 4-OH), 8.85 (s, 1H, 5^′^-OH), 7.78 (d, 2H, *J*_2,3_ = *J*_6,5_ = 8.4 Hz, H-2, H-6), 6.95 (s, 1H, H-6^′^), 6.87 (d, 2H, *J*_3,2_ = *J*_5,6_ = 8.8 Hz, H-3, H-5), 6.71 (s, 2H, H-3^′^, H-4^′^), 3.00 (q, 2H, *J* = 13.6 Hz, 6.8 Hz, CH_2_), 1.14 (t, 3H, *J* = 7.2, CH_3_); EI-MS: *m/z* (rel. abund. %), 300 (M^+^, 98), 283 (18), 271 (92), 135 (28), 121 (100), 93 (59); Anal. Calcd for C_16_H_16_N_2_O_4_: C, 63.99; H, 5.37; N, 9.33; O, 21.31; Found: C, 63.97; H, 5.38; N, 9.30.

## Results and discussion

### Chemistry

For the study of thymidine phosphorylase inhibitory activity, twenty-nine derivatives of 4-hydroxybenzohydrazides were synthesized by reacting 4-hydroxybenzohydrazide with substituted benzaldehydes in ethanol, catalyzed by acetic acid (Scheme 1).

### Scheme 1. Synthesis reaction: Synthesis of 4-hydroxybenzohydrazides 1–29

**Fig 2:**
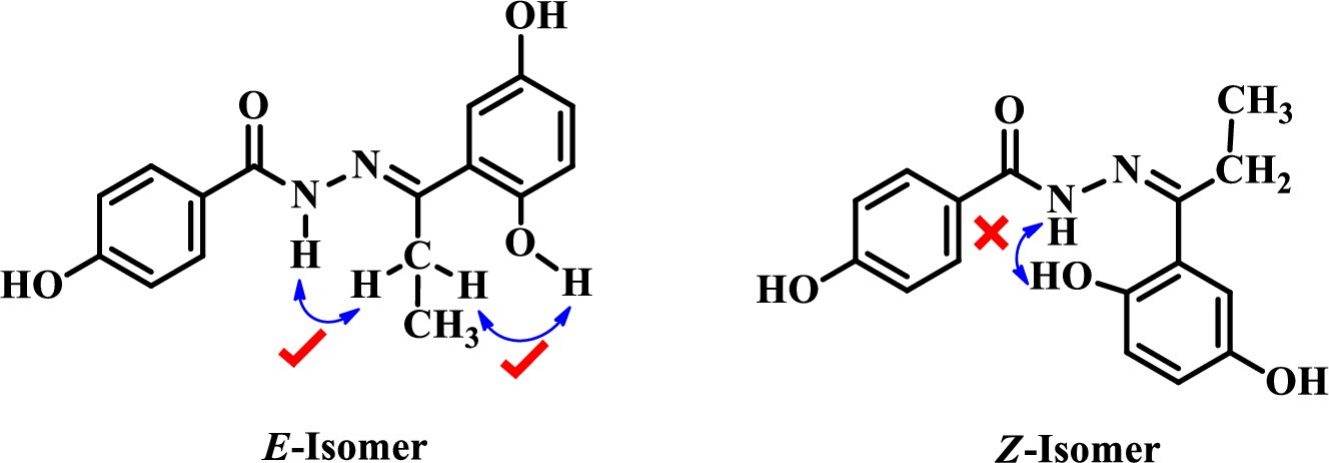
NOESY interaction: Distinctive NOESY interactions to confirm E/Z configuration.

The structures of these derivatives were identified by EI-MS, and ^1^H-NMR spectroscopy, and comparison with the data reported in literature. To confirm the stereochemical assignment of iminic bond, NOESY (nuclear overhauser enhancement spectroscopy) spectrum was recorded for a representative compound **29**. Strong NOESY interactions of NH and 2^′^-OH with CH_2_ of ethyl group revealed the geometry of the compound to be of (*E*)-configuration. Absence of NOESY interaction of NH with 2^′^-OH further confirmed the *E* stereochemistry (Fig 2).

### Enzyme inhibition studies

**Fig 3:**
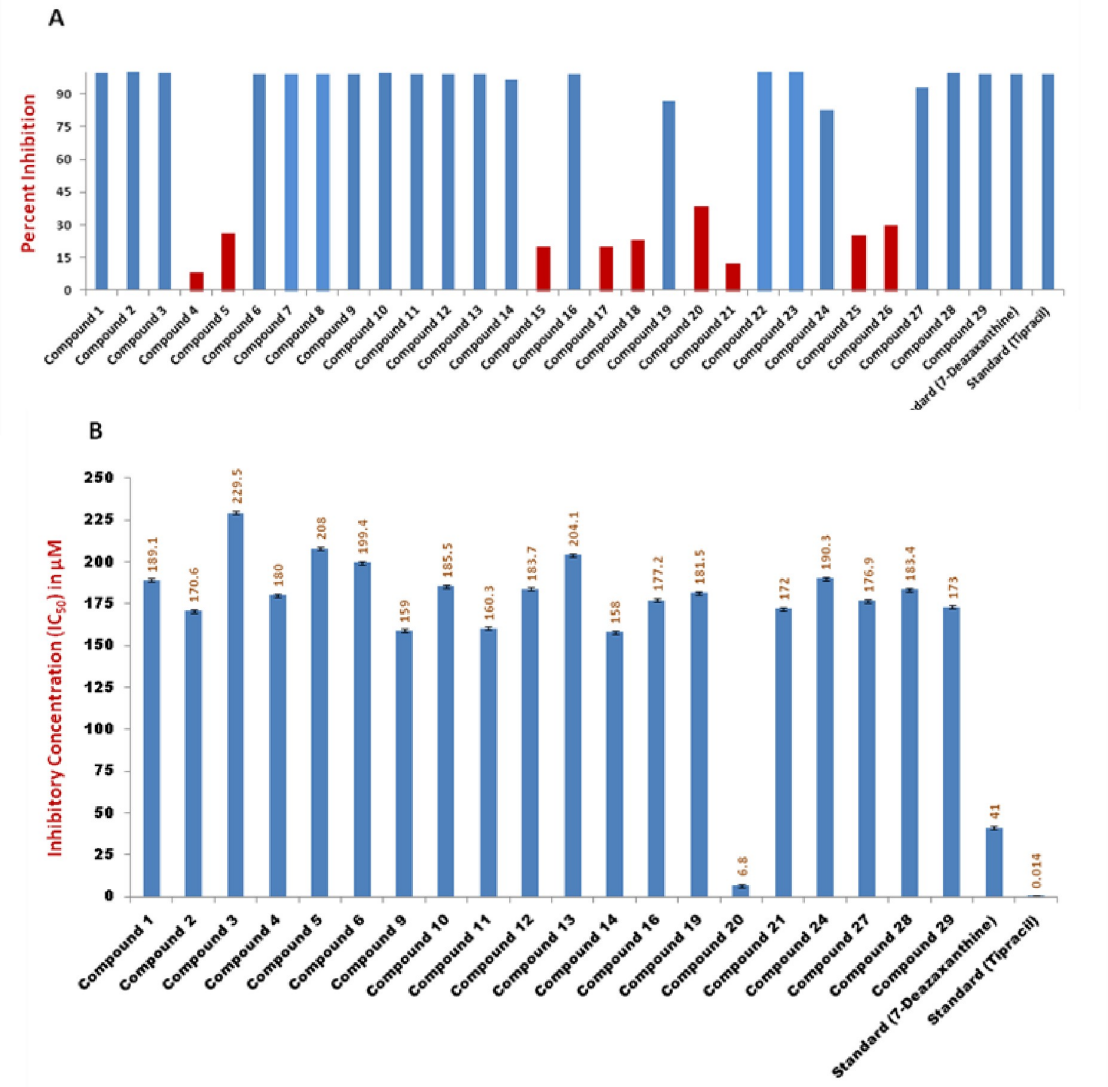
***In-vitro* thymidine phosphorylase inhibitory activities of 4-hydroxylbenzohydarazides (1–29):** (A) Percent inhibition, blue color bars represents active compounds (compounds which showed ≥50%), while red color represent inactive compounds (compounds which showed < 50% inhibition) (B) IC_50_ values of active compounds is given in *μ*M.

**Fig 4:**
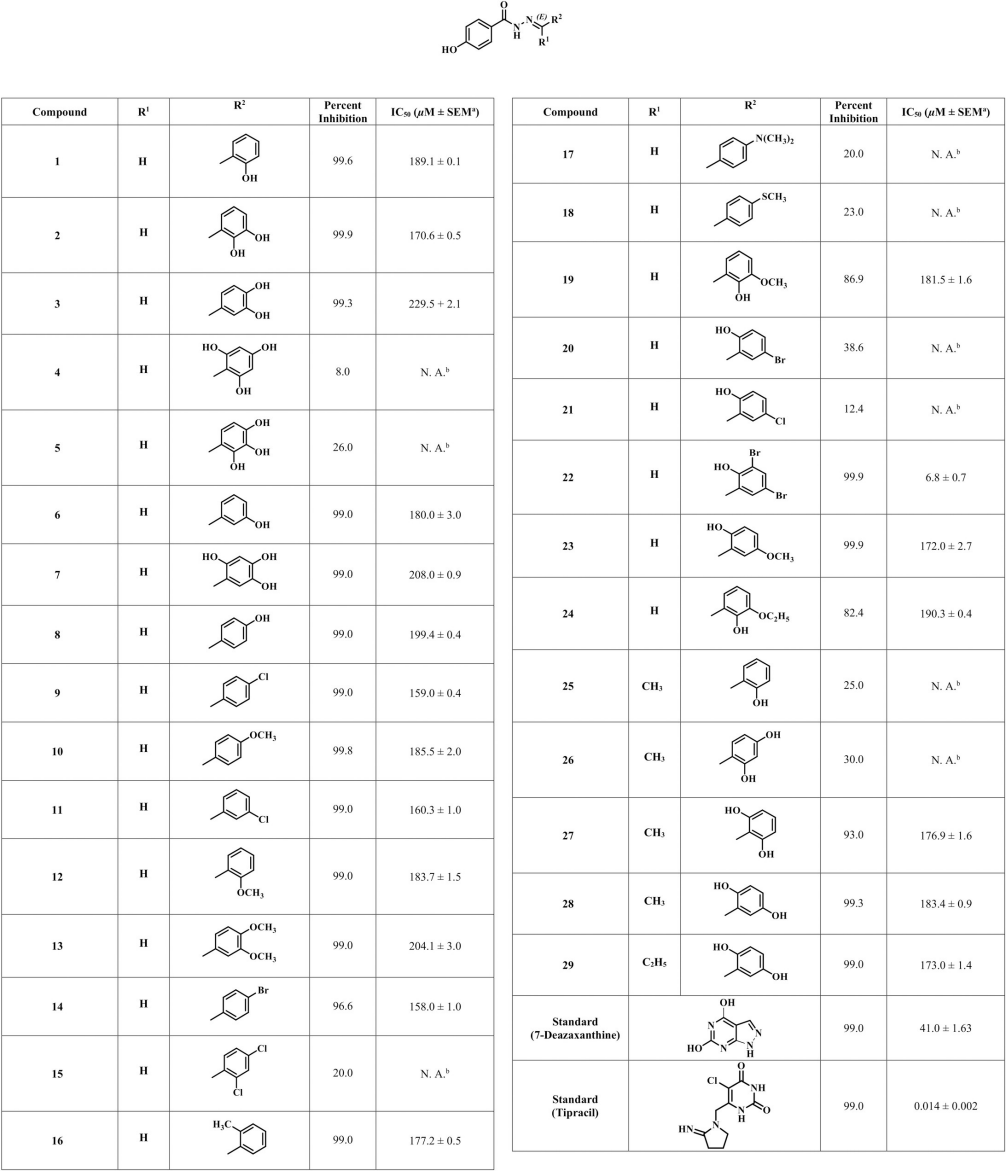
*In-vitro* thymidine phosphorylase inhibitory activities of 4-hydroxylbenzohydarazides (1–29).

Twenty-nine analogues of 4-hydroxybenzohydarzides (**1**–**29**) were evaluated for TP inhibition, in comparison to the standards *i*.*e*. tipracil (IC_50_ = 0.014 ± 0.002 *μ*M) and 7-deazaxanthine (IC_50_ = 41.0 ± 1.63 *μ*M). Among them, twenty compounds (**1**–**3**, **6**–**14**, **16**, **19**, **22**–**24**, and **27**–**29**) were found to be active against TP. Compound **22** was the most active compound of the series with IC_50_ = 6.8 ± 0.7*μ*M, while other compounds showed a moderate inhibition of TP (Fig 3, Fig 4). In Fig 4 and Tables, SEM^a^ stands for standard error of mean, while N.A^b^ represent not active.

Eight derivatives with mono-, di- and tri-hydroxy substitutions were evaluated, and six compounds **1**–**3**, **6–8** were found to be active against TP with IC_50_ values of 170.6 to 229.5 *μ*M. Among them, compound **2** with two OH groups at *ortho* (C-2′) and *meta* (C-3′) positions showed a moderate TP inhibition (IC_50_ = 170.6 ± 0.5 *μ*M), followed by compound **6** (IC_50_ = 180.0 ± 3.0 *μ*M) with a single OH group at *meta* (C-3′) position. Tri-hydroxylated derivatives **4**–**5** showed 8 and 26% inhibition, respectively (Fig 4).

Three derivatives with mono-, and di-methoxy substitutions (compounds **10**, **12**, **13**) were evaluated, and found to be weak TP inhibitors (IC_50_ = 183.7 ± 1.5, 185.5 ± 2.0, and 204.1 ± 3.0 *μ*M, respectively). Among them compound **12** with a OCH_3_ group at *ortho* position showed a moderate TP inhibition (IC_50_ = 183.7 ± 1.5 *μ*M).

Empirical structure-activity relationship (SAR) studies proposed that hydroxy and methoxy substitutions on phenyl ring play an important role in inducing TP inhibition. These groups may be involved in hydrogen bonding with the amino acid residues, present at the substrate-binding site or hydrophobic pocket of TP enzyme.

Four halogen-substituted derivatives were evaluated (compounds **9**, **11**, **14**, **15**), and three were found to inhibit the TP activity with IC_50_ values between 158.0–160.3 *μ*M. Among these mono-halogenated derivatives, **9**, **11**, and **14** showed ability to inhibit the enzyme activity, while di-halogenated derivative **15** was found to be inactive with 20% inhibition. Based on the IC_50_ values, halogens substitution were found to be more favorable in comparison to OH and OCH_3_ substitutions. It was thus proposed that halogens might increase the ability of these compounds to interact *via* hydrogen bonding with amino acid residues present at substrate binding-site of TP.

Compound **16** with a methyl group at *ortho* position showed a moderate TP inhibition (IC_50_ = 177.2 ± 0.5 *μ*M), while compounds **17**, and **18** with dimethylmine and methylsulfanyl groups, attached to *para* position, respectively showed less than 50% inhibition thus regarded inactive.

Three derivatives with hydroxyl-*cum*-methoxy substitutions **19**, **23**, **24** were evaluated, and all were found to be moderately active against TP enzyme (IC_50_ values between 172.0–190.3 *μ*M). Compound **19** with OH and OCH_3_ groups at *ortho* (C-2′) and *meta* (C-3′) positions, respectively, showed a moderate TP inhibition (IC_50_ = 181.5 ± 1.6 *μ*M). Switching of hydroxy group to the other *meta* (*i*.*e*. C-5′) position, as in compound **23**, slightly increased the inhibition of TP (IC_50_ = 172.0 ± 2.7 *μ*M). Replacement of methoxy with an ethoxy group, as in compound **24**, slightly decreased the TP inhibitory activity (IC_50_ = 190.3 ± 0.4 *μ*M). SAR proposed that when hydroxyl and methoxy groups are present adjacent to each other, they lower the ability of compounds to inhibit enzyme, as inferred from their IC_50_ values.

Three derivatives with hydroxy-*cum*-halogen substitutions were also evaluated (compounds **20**–**22**). Among them compound **22** with two bromo groups at *meta* (C-3′ and C-5′) positions, and an OH at *ortho* (C-2′) showed a potent TP inhibitory activity (IC_50_ = 6.8 ± 0.7 *μ*M), in comparison to the standard used *i*.*e*., 7-deazaxanthine (IC_50_ = 41.0 ± 1.63 *μ*M). This is an unusual but reproducible behavior exhibited by this compound, whereas compound **20**, which just lacks a bromo group at *meta* (C-3′) position showed 38.6% inhibition. As TP has hydrophobic pocket near the substrate binding sites, it is possible that compound **22** with *di*-bromo substitution may be able to fit more appropriately at the hydrophobic pocket of the TP, which may not be possible for compound **20**. Compound **20** might have a conformation which does not fit well in the hydrophobic pocket of enzyme.

Derivatizations were also made by replacing the benzylidene group with ethylidine and propylidine groups (Fig 1), in addition to OH substitutions on phenyl ring. Five derivatives **25**–**29** were evaluated, and among them compounds **27**, **28**, and **29** with two OH groups on phenyl ring, showed a moderate inhibition of TP (IC_50_ = 176.9 ± 1.6, 183.4 ± 0.9, and 173.0 ± 1.4*μ*M, respectively). This is consistent with the results we obtained for dihydroxylated derivatives with benzylidine group (Fig 4).

### Kinetic studies

**Fig 5:**
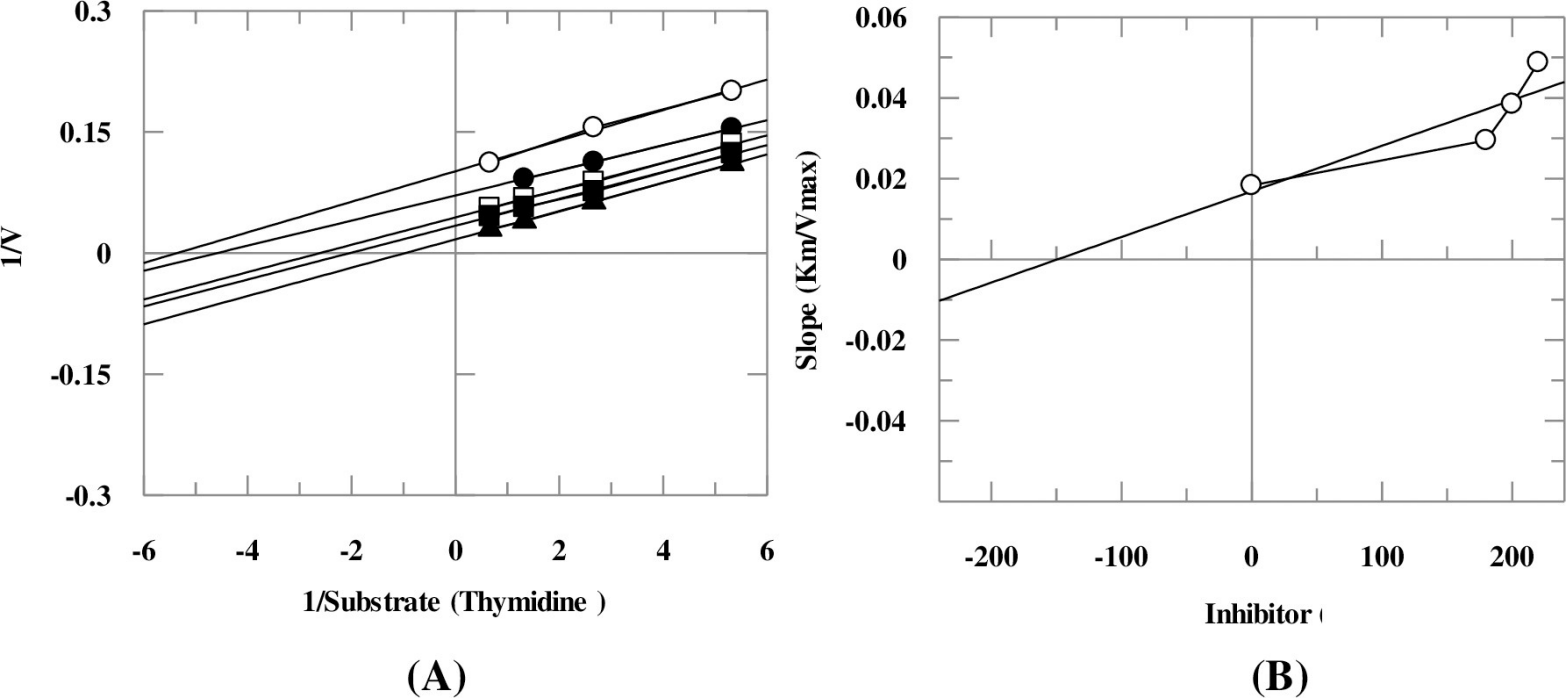
**Kinetic mode of compound 27** (A) Depicts Lineweaver-Burk plot of compound **27** in which reciprocal of substrate concentration (1/*S*) is plotted on *x*-axis, while reciprocal of rate of reaction (1/*V*) is plotted on *y*-axis in the absence and present of different concentrations of compound **27**. **(B)** Depicts secondary re-plot of Lineweaver-Burk plot between the slopes (*K*_m_/*V*_max_) of each line *versus* different concentrations of compound **27**.

**Fig 6:**
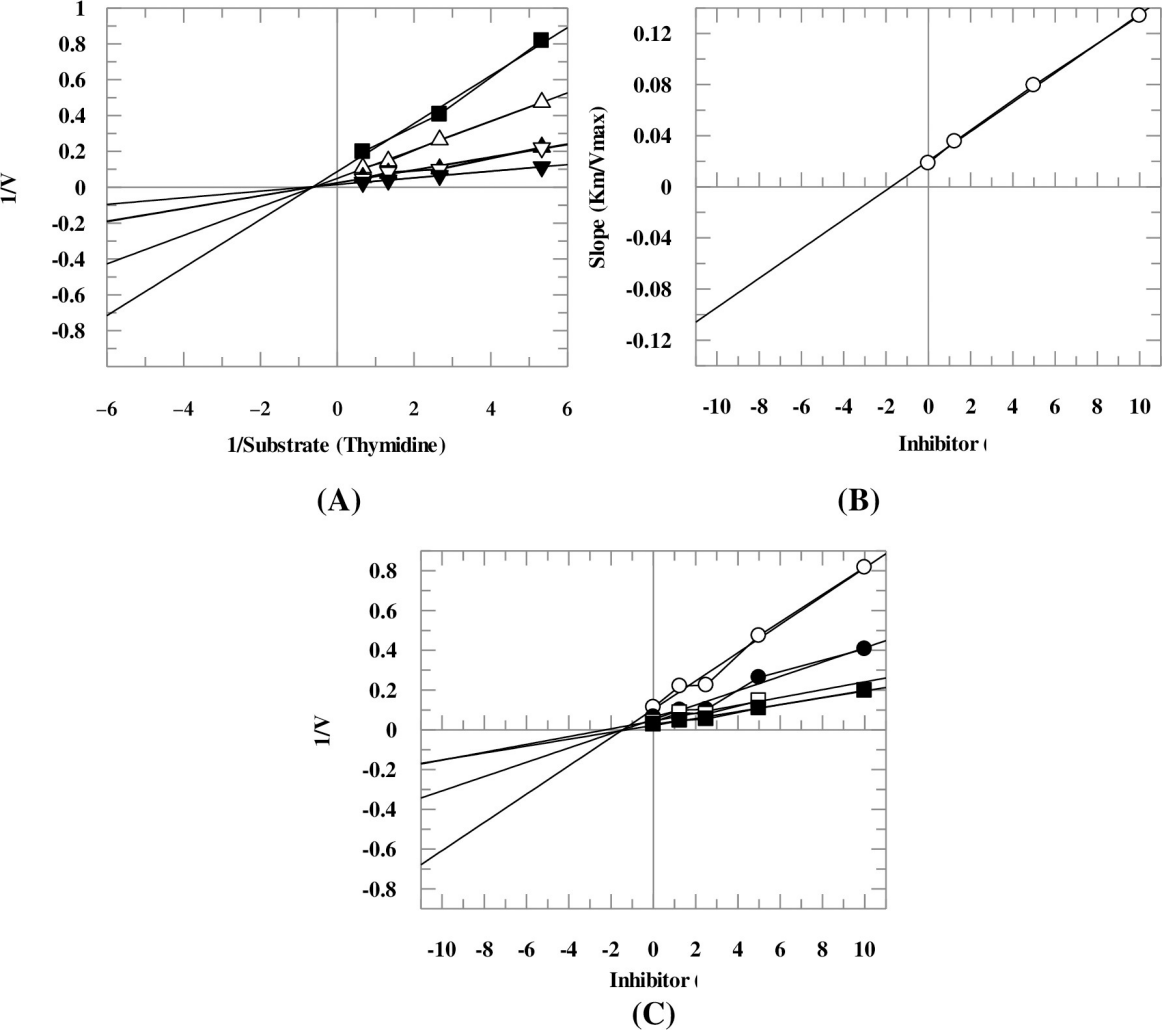
**Kinetic mode of compound 22** (A) Depicts Lineweaver-burk plot of compound **22** in which reciprocal of substrate concentration (1/*S*) is plotted on *x*-axis, while reciprocal of rate of reaction (1/*V*) is plotted on *y*-axis in the absence and presence of different concentrations of compound **22**. Figure shows that apparent km of the enzyme remains unaffected while the apparent *V*_max_ decreased. (**B**) Depicts secondary re-plot of Lineweaver-Burk plot between the slopes (*K*_m_/*V*_max_) of each line *versus* different concentrations of compound **22**. (**C**) Depicts Dixon plot of reciprocal of rate of reaction (velocities) *versus* different concentrations of compound **22**.

Kinetic study on compounds **3**, **9**, **14**, **27**, and **29** revealed that they inhibit the TP in an uncompetitive manner (Table 1), as deduced from the Lineweaver-Burk plot. Uncompetitive inhibitors interact with enzyme only when enzyme-substrate (ES) complex is formed. ES complex formation was proposed to induce conformational changes in the enzyme which facilitates the binding of the inhibitor. Uncompetitive inhibitors cause decrease in both *K*_m_ and *V*_max_ values of the enzyme (Fig 5). Compound **22** inhibited the enzyme in a non-competitive manner (Fig 6). This compound, therefore, interacted either with the amino acids of hydrophobic pocket of the enzyme or at allosteric site of the enzyme. Noncompetitive inhibitors do not affect the *K*_m_ value but changes the *V*_max_ value. These compounds, therefore, do not competitively interact with the thymidine or phosphate-binding sites of TP when thymidine is used as the variable substrate. Values of dissociation constants (*K*i) were determined by secondary re-plot of Lineweaver-Burk plot, and Dixon plot, these were in the range of 1.75–176 *μ*M (Table 1).

**Table 1:** Kinetic studies of most active compounds on thymidine phosphorylase.

Compound	*K*_i_ (*μ*M ± SEM)^a^	Inhibition Type
**3**	176.65 ± 0.006	Uncompetitive
**9**	138.0 ± 0.009	Uncompetitive
**14**	80.5 ± 0.002	Uncompetitive
**22**	1.75 ± 0.009	Non-competitive
**27**	168.0 ± 0.003	Uncompetitive
**29**	159.05 ± 0.002	Uncompetitive
**7-Deazaxanthine**	45.66 ± 0.0009	Non-competitive

### Molecular docking studies

**Fig 7:**
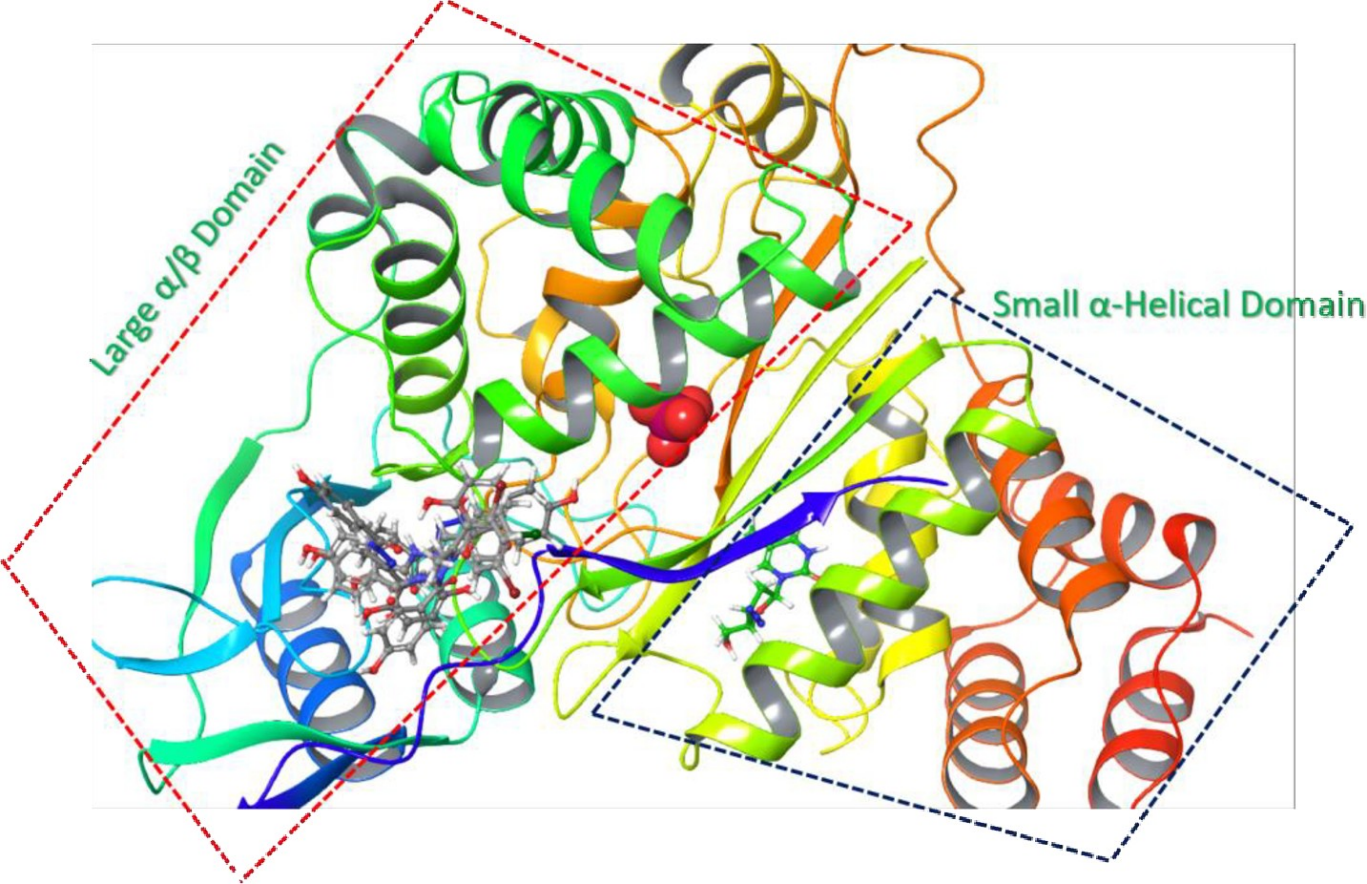
**Ribbon representation of TP (PDB:4LHM): It is showing a large *α*/*β* domain (red dotted lines) and a small *α*-helical domain (blue dotted lines).** The inhibitor AZZ (green ball and stick) occupies the nucleoside binding pocket of TP while the phosphate binding site is in the *α*/*β* domain of TP. Compounds **3**, **9**, **14**, **22**, **27**, and **29** (grey ball and stick) binds in the *α*/*β* domain of TP.

Molecular docking studies on selected inhibitors were performed in order to understand the ligand-receptor interactions at atomic level. Compounds **3**, **9**, **14**, **22**, **27**, and **29** were found to be either uncompetitive or non-competitive inhibitors of TP. They showed binding to an allosteric site, located adjacent to the substrate binding site of thymidine phosphorylase. According to the crystal structure of TP (4LHM), it has a small α-helical domain containing residues from 1 to 65, 163 to 193 that makes the thymidine binding site. While, a large *α*/*β* domain with residues 80 to 154, 197 to 440 make the phosphate binding site. These two domains are separated by a large cleft, and the movement of these two domains brings the two substrate binding sites closer for the initiation of the catalytic activity. There are several homologous flexible loops located at different positions of TP, comprising of 173–178, 206–213, 367–381 amino acid residues [44,45]. The molecular docking analysis of selected inhibitors showed that most of them were able to interact with the amino acid residues of large *α*/*β* domain (197 to 440) which play an important role in the catalytic activity of TP (Fig 7).

**Fig 8:**
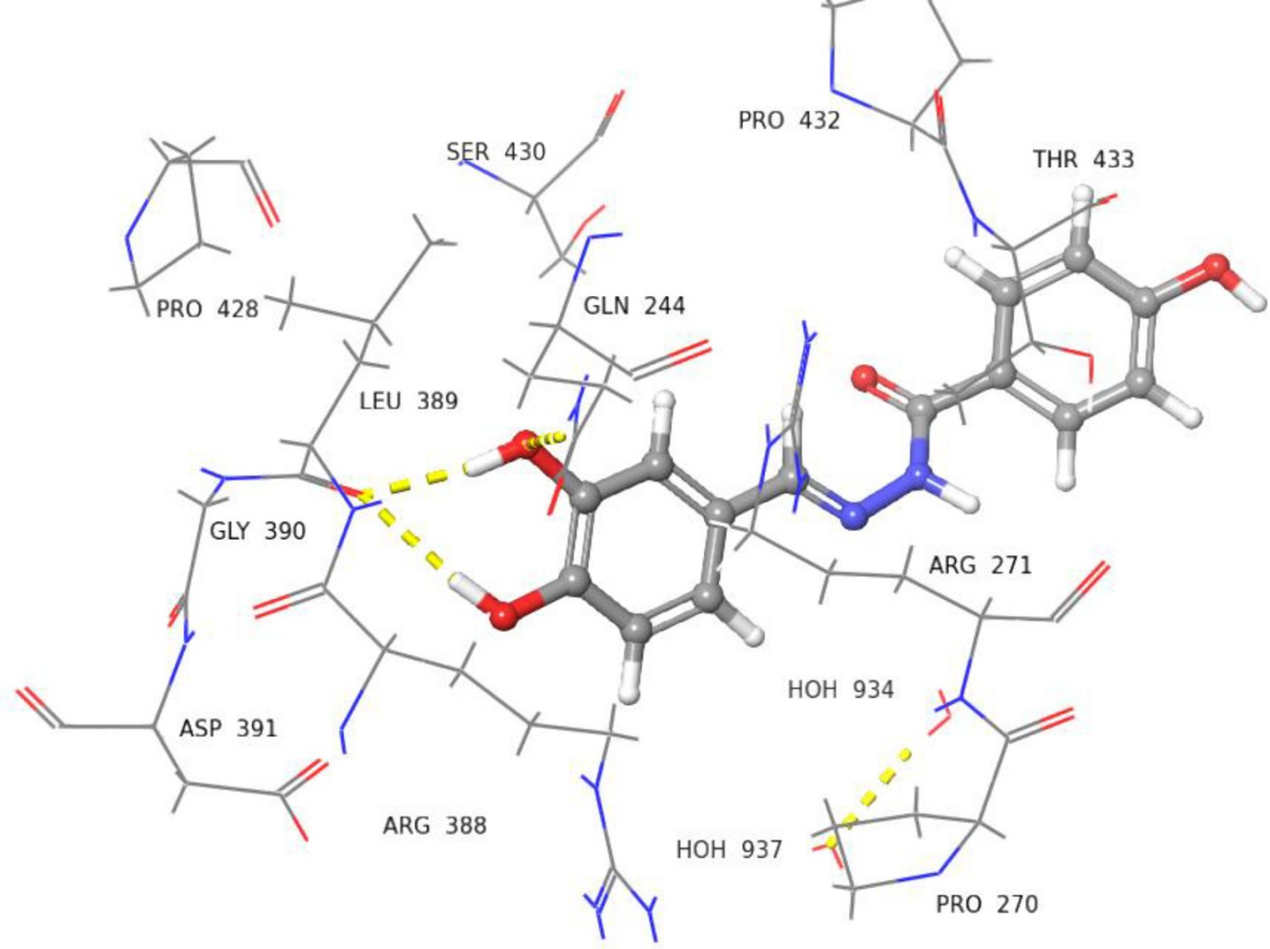
3D Representation of docked pose of compound 3 (IC_50_ = 229.5 ± 2.1 *μ*M). The two OH groups showed H-bonding with Leu389 and Gln244 (yellow dotted lines).

**Fig 9:**
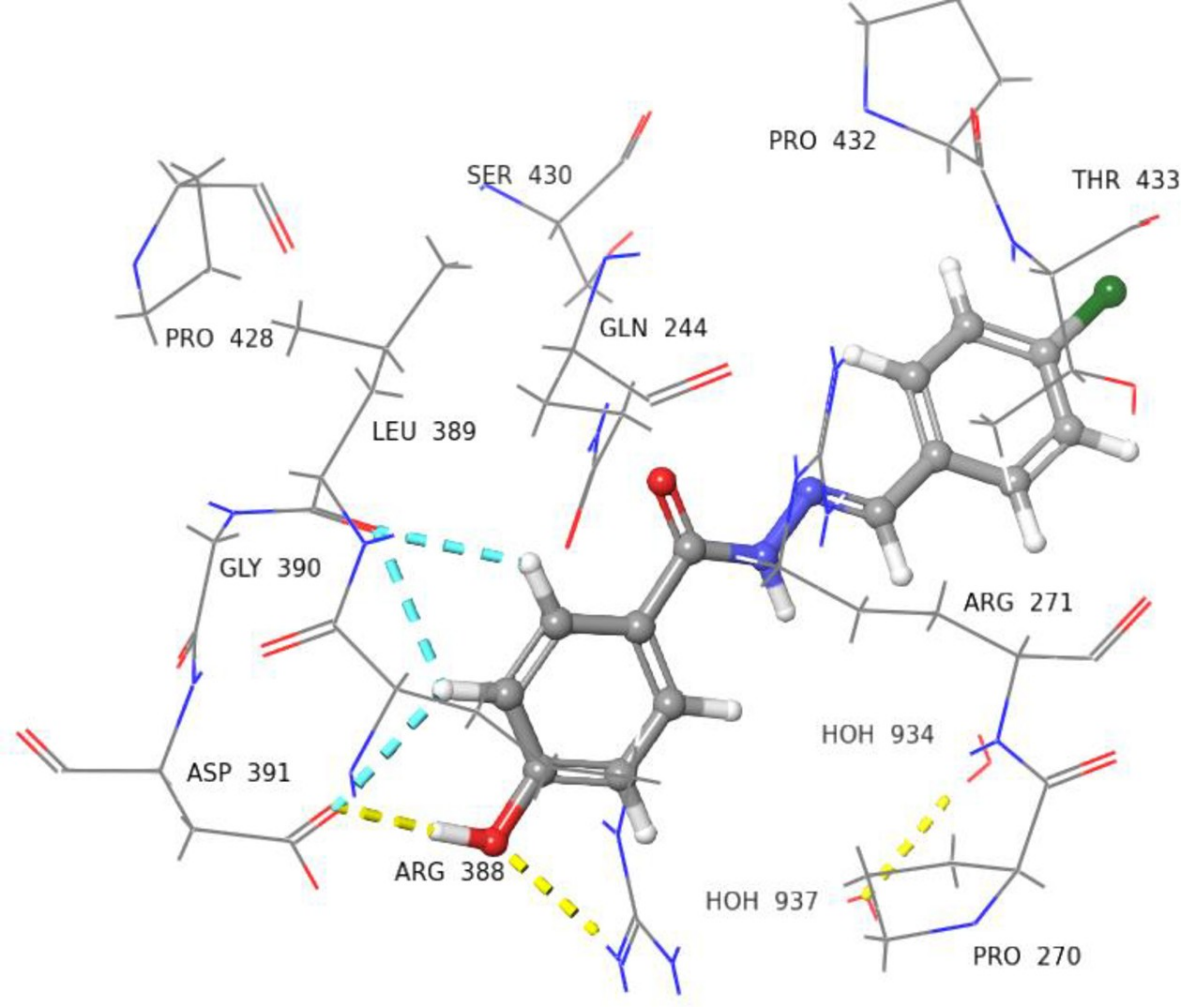
**3D Representation of docked pose of compound 9 (IC_50_ = 159.0 ± 0.4 *μ*M): It is showing interaction with the Asp391, and Arg388 *via* H-bonds (yellow and blue dotted lines)**.

**Fig 10:**
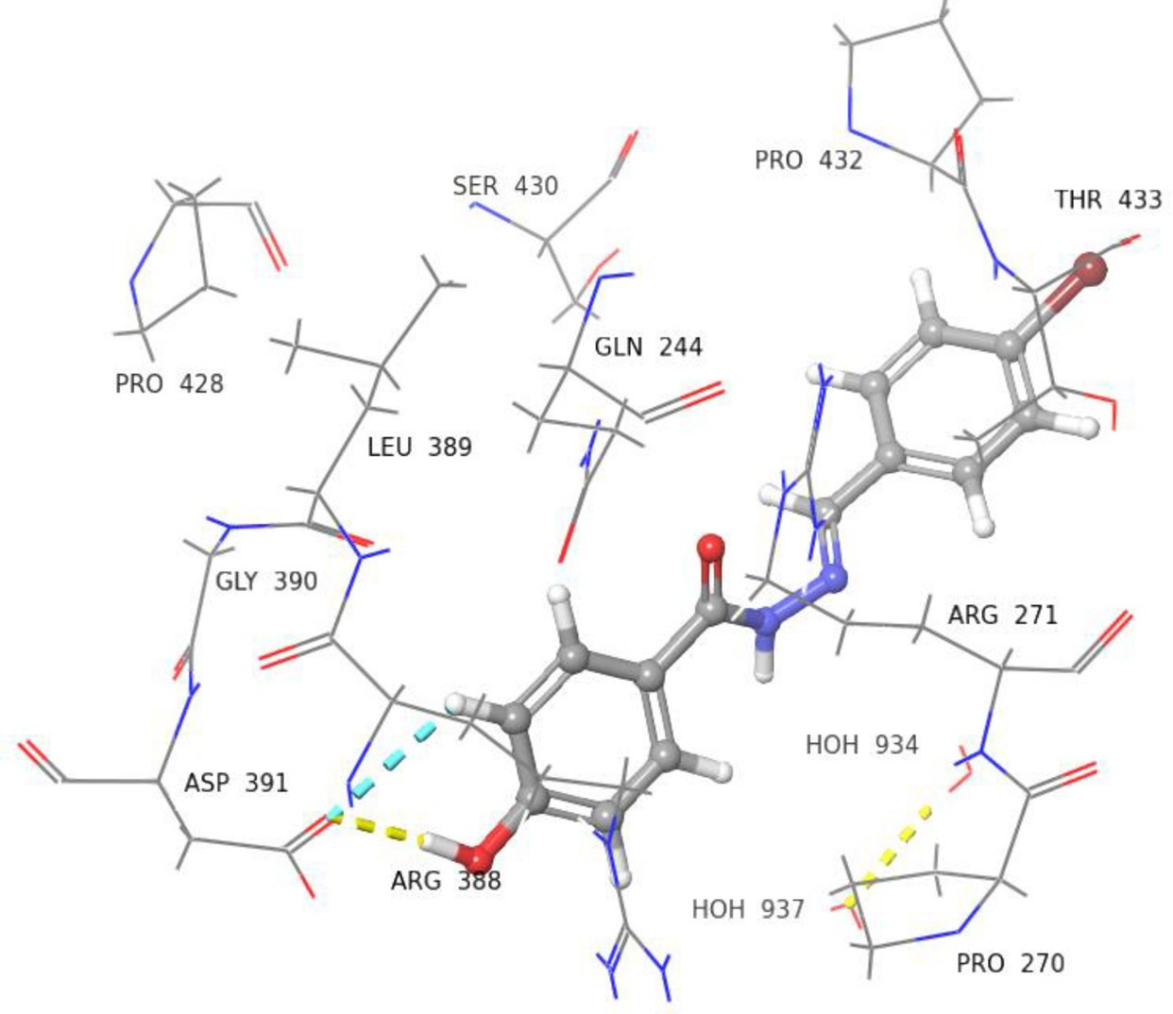
3D Representation of docked pose of compound 14 (IC_50_ = 158.0 ± 1.0 *μ*M). It is showing interaction with Asp391 *via* H-bonds (yellow and blue dotted lines).

**Fig 11:**
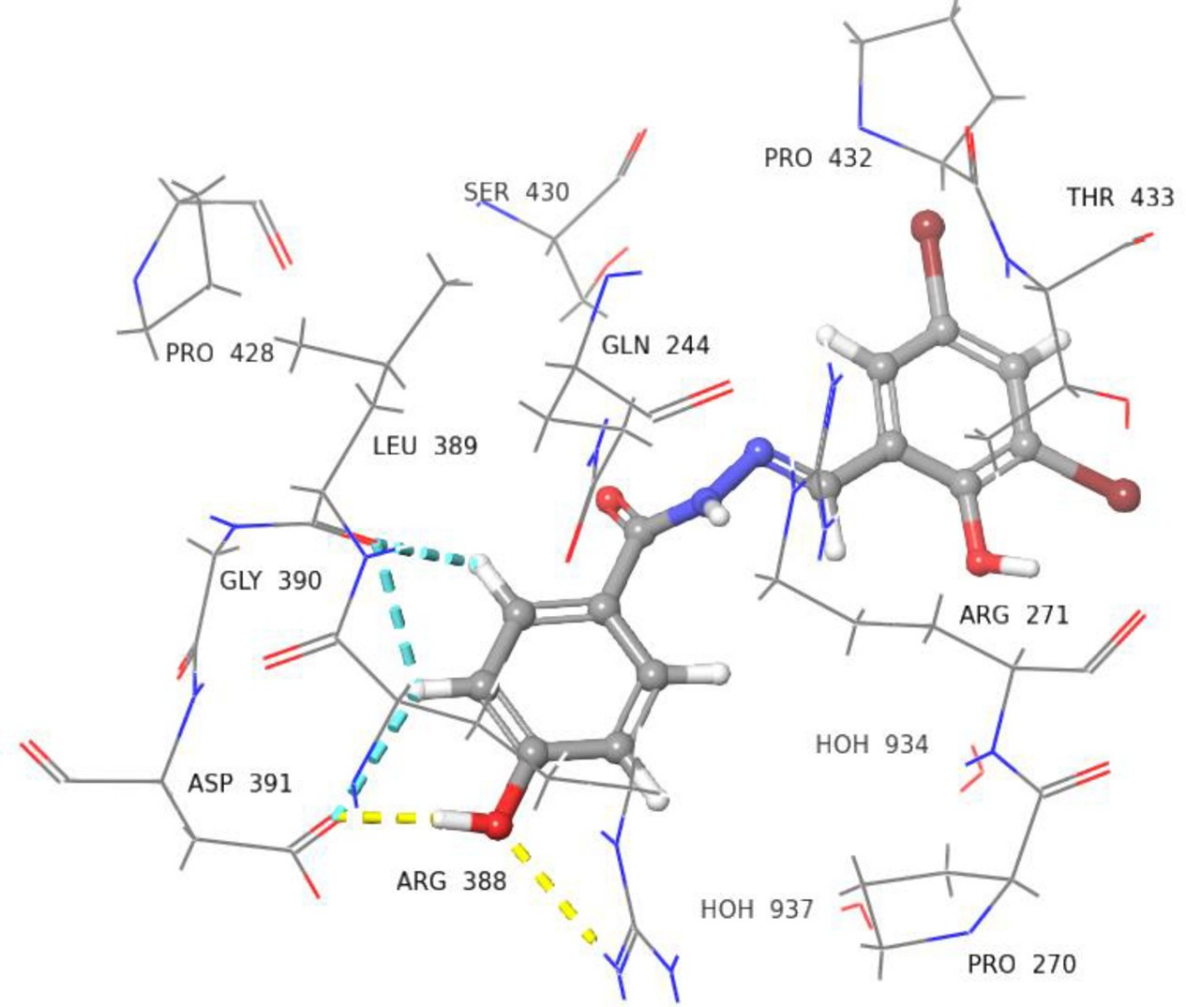
3D Representation of docked pose of compound 22 (IC_50_ = 6.8 ± 0.7 *μ*M). The OH group is interacting with Asp391 *via* H-bond (yellow dotted lines), while the hydrogens of phenyl ring are interacting with Leu389 *via* H-bonds (blue dotted lines).

Compound **5** with dihydroxyl groups at *meta* and *para* positions were found to interact with Leu389 and Gln244 *via* H-bonds (Fig 8). Compounds **9**, **14**, and **22** showed slightly different docked poses, in comparison to compound **5**. For instance, the OH group of phenyl ring in compound **9** was able to form H-bonds with Asp391, and Arg388 (Fig 9). Compound **14** was able to form H-bond interactions with Asp391 (Fig 10). Similarly, compound **22** was also able to interact with Asp391, Arg388, and Leu389 *via* H-bonds (Fig 11).

**Fig 12:**
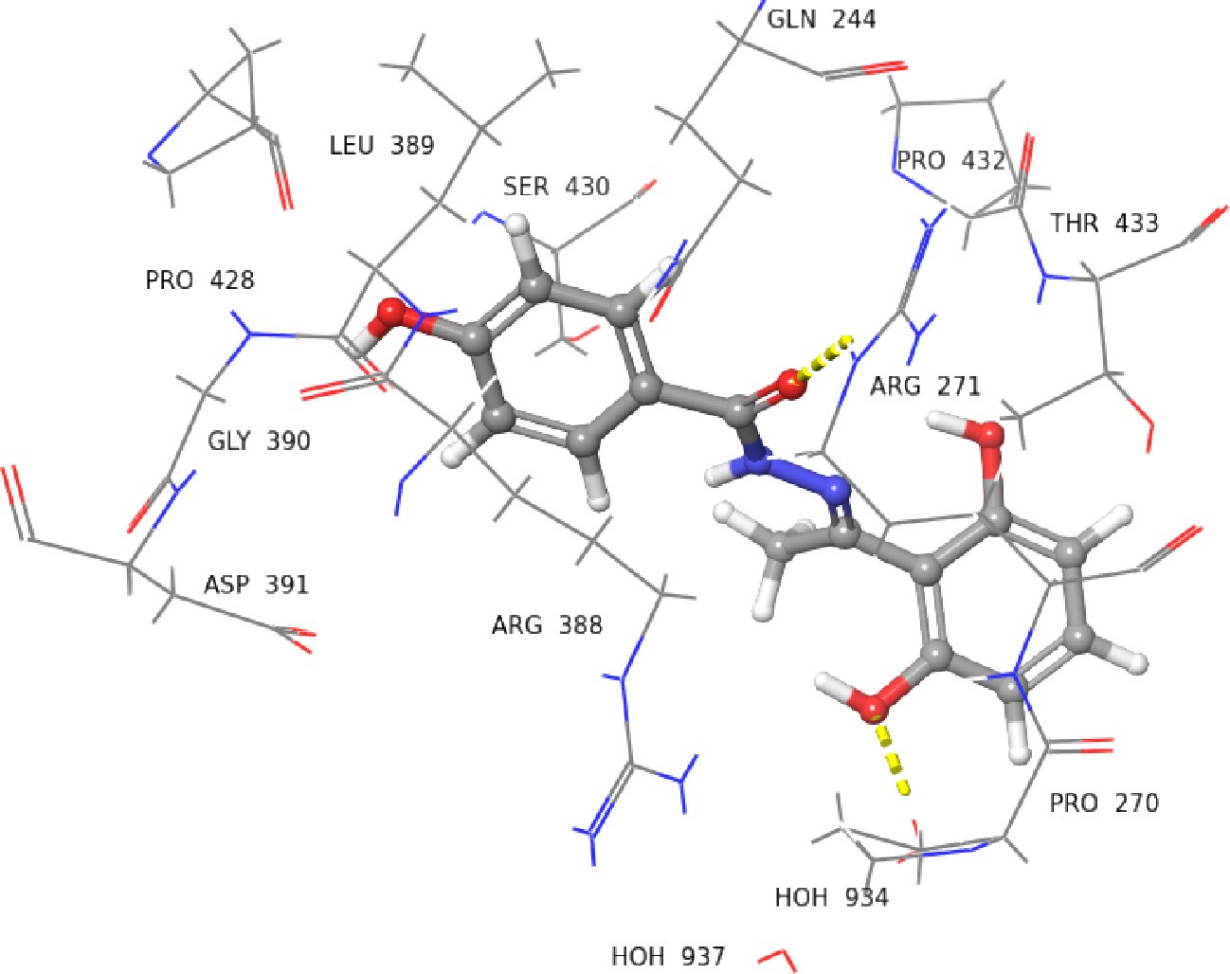
3D Representation view of docked pose of compound 27 (IC_50_ = 176.9 ± 1.6 μM). The carboxyl group of hydrazide is interacting with Arg271 *via* H-bond (yellow dotted lines), while the *ortho* substituted OH group is interacting with Pro270 *via* H-bond (blue dotted lines).

**Fig 13:**
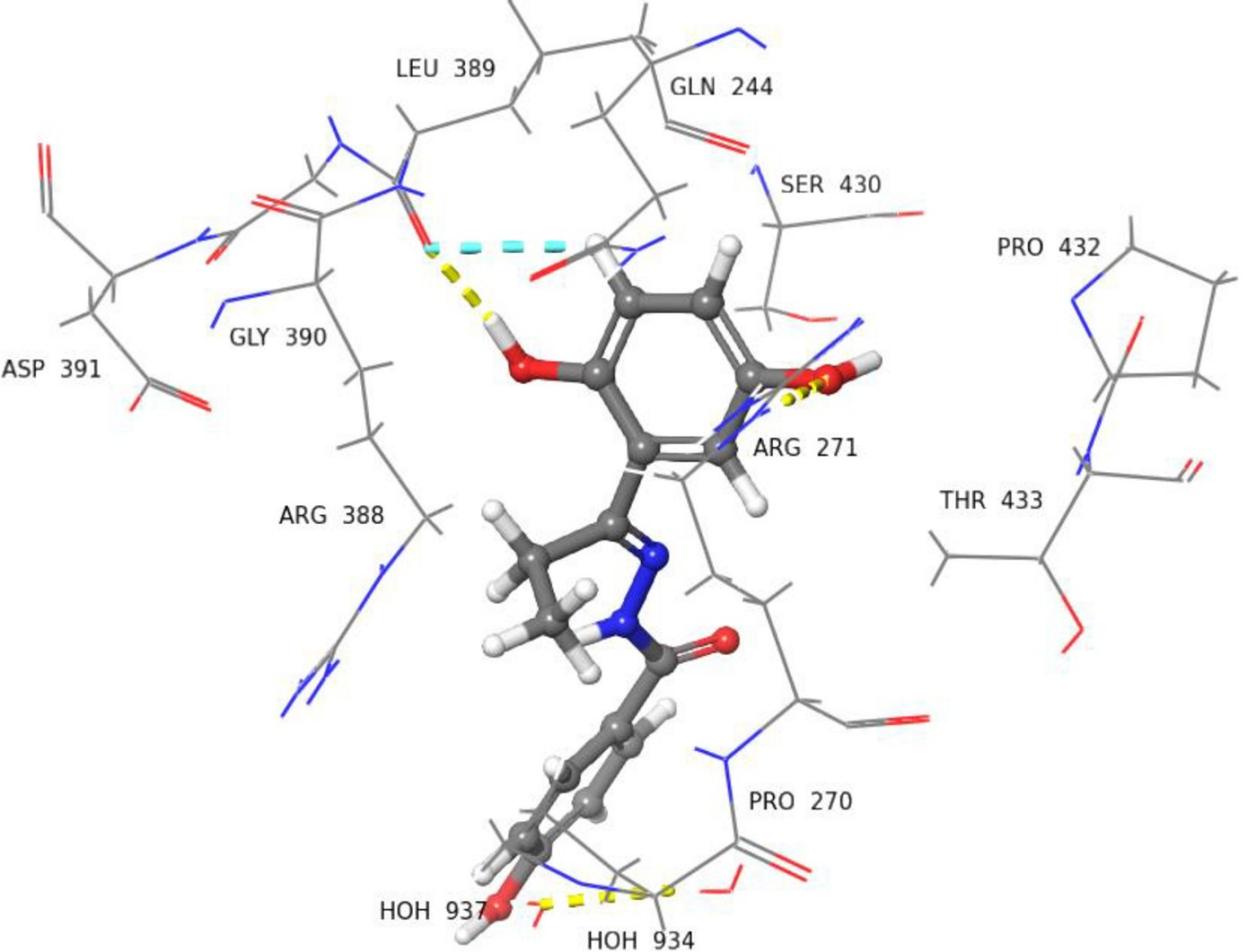
3D Representation of docked pose of compound 29 (IC_50_ = 173.0 ± 1.4 *μ*M). The *ortho* substituted OH group is interacting with the Leu389 *via* H-bond (yellow dotted lines), and the phenyl ring hydrogen is interacting with Leu389 *via* H-bond (blue dotted lines). The *meta* substituted OH group is interacting with Arg271 *via* H-bond.

Compounds **27**, and **29** possess CH_3_ and C_2_H_5_, respectively, as R_2_ substituents in addition to the hydroxyl group substituted phenyl ring. These alkyl groups further changed the docked poses of compounds **27**, and **29**. For instance, in compound **27** the carboxyl group of hydrazide was found to be involved in interacting with Arg271 and the OH group of phenyl ring interacted with water molecule *via* H-bond (Fig 12). The *ortho* substituted hydroxyl group in compound **29** interacted with carboxyl group of Leu389 *via* H-bond (Fig 13), while the *meta* substituted OH group interacted with the side chain of Arg271 *via* H-bond.

### Anti-proliferative studies

Compounds found to be active against TP enzyme were then subjected to MTT proliferation assay. Rapid proliferation is the property inherent to cancerous/ tumor cells. TP is reported to be highly expressed in prostate cancer. Therefore, the compounds active against TP were evaluated for their anti-proliferative effect on prostate cancer (PC3) cells, and cytotoxic effect on mouse fibroblast (3T3) cells [2, 30, 31].

Compounds **1**, **2**, **3**, **7**, **19**, **22–24**, and **27**–**29** were found cytotoxic towards the proliferation of 3T3 cells with IC_50_ values in between 3.8–26.7 *μ*M, in comparison to the reference compound *i*.*e*. cyclohexamide (IC_50_ = 0.26 ± 0.1 *μ*M). Compounds **1**, **2**, **19**, and **22–24** inhibited the proliferation of PC3 cells with IC_50_ values in between 6.5–10.5 *μ*M, in comparison to the reference drug *i*.*e*. doxorubicin (IC_50_ = 0.91 ± 0.1 *μ*M) (Table 2).

**Table 2:** *In-vitro* anti-proliferative activities of active compounds.

Compound	IC_50_ ± Standard Deviation (*μ*M)	
	3T3 Cell Line	PC3 Cell Line
**1**	13.1 ± 0.3	7.5±0.4
**2**	23.0 ±1.0	7.6±0.4
**3**	23.5 ± 1.1	>30
**6**	>30	>30
**7**	3.8 ± 0.4	>30
**8**	>30	>30
**9**	>30	>30
**10**	>30	>30
**11**	>30	>30
**12**	>30	>30
**13**	>30	>30
**14**	>30	>30
**16**	>30	>30
**19**	23.6 ± 0.2	9.6±0.3
**22**	9.2 ± 0.1	8.3±0.9
**23**	15.3 ± 0.9	10.5±0.5
**24**	9.4 ± 0.3	6.5 ±0.2
**27**	26.7 ± 1.0	>30
**28**	20.2 ± 0.9	>30
**29**	19.5 ± 0.9	>30
**Standard Cyclohexamide)**	**0.26 ± 0.1** *μ***M**	-
**Standard (Doxorubicin)**	-	**0.91± 0.1** *μ***M**

IC_50_ Values of compounds **1**, **2**, **19**, and **22**–**24** for cytotoxicity activity is lower in cancerous cells (IC_50_ = 7.5 ± 0.4, 7.6 ± 0.4, 9.6 ± 0.3, 10.5 ± 0.5, 6.5 ± 0.2, and 8.3 ± 0.9 *μ*M, respectively) than on normal cells (IC_50_ = 13.1 ± 0.3, 23.0 ±1.0, 23.6 ± 0.2, 15.3 ± 0.9, 9.4 ± 0.3, and 9.2 ± 0.1 *μ*M, respectively). These compounds therefore possess dual characteristics as they can inhibit the TP enzymatic activity, and proliferation of PC3 cells. Their dual inhibitory potential deserves to be studied further for anticancer activity.

Compounds which showed anti-proliferation activity only towards 3T3 cell lines (such as **3**, **7**, **27**–**29**) were cytotoxic to normal cells and therefore need to be investigated again for above-mentioned activities after structural modification.

## Conclusion

Role of TP in inducing angiogenesis and tumor growth makes it an important target for the discovery of anti-angiogenic (anti-cancer) agents. In this regards, a series of 4-hydroxybenzohydrazides (**1–29**) was evaluated for its *in-vitro* inhibitory activity against angiogenic enzyme TP. Twenty derivatives were found to inhibit the TP enzymatic activity. Among them, compound **22** showed a several fold more potent TP inhibitory activity (IC_50_ = 6.8 ± 0.7 *μ*M), when compared to the standard, 7-deazaxanthine (IC_50_ = 41.0 ± 1.63 *μ*M). In mechanistic studies, it showed a non-competitive mode of inhibition (*K*i = 1.75 ± 0.009 *μ*M). Compound **22** apparently interact with Arg271 and Pro270 of enzyme *via* H-bonds. Furthermore, it also showed a good anti-proliferative (cytotoxic) activity against prostate cancer (PC3) cell line. Present study thus identifies a new class of inhibitors against TP enzyme, and cancer cells proliferation. This class can be investigated further for anti-cancer studies at *in-vivo* level.

## Supporting information

S1 Fig (TIF)
