# Supplementary figures and images for: Thymidine phosphorylase and prostrate cancer cell proliferation inhibitory activities of synthetic 4-hydroxybenzohydrazides: In vitro, kinetic, and in silico studies
Source: PLoS One. 2020 Jan 27;15(1):e0227549. doi: 10.1371/journal.pone.0227549 (PMC6984732; doi:10.1371/journal.pone.0227549)

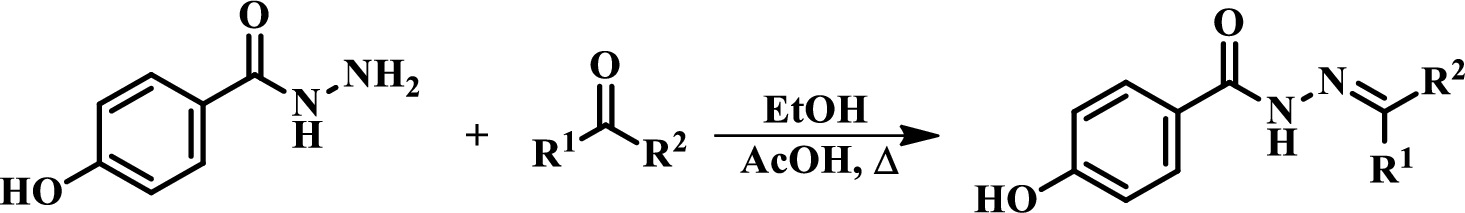

Supplement: S1 Fig — (TIF) [file pone.0227549.s001.tif]
